# Supplementary material for: Genome anchoring, retention, and release by neck proteins of Staphylococcus phage 812
Source: Commun Biol. 2026 Jan 8;9:199. doi: 10.1038/s42003-025-09477-8 (PMC12886913; doi:10.1038/s42003-025-09477-8)
Supplement: Supplementary file 2 — Supplementary Information [file 42003_2025_9477_MOESM2_ESM.pdf]

Supplementary Information

Genome anchoring, retention, and release by neck proteins of *Staphylococcus* phage 812

Zuzana Cieniková, Jiří Nováček, Marta Šiborová, Barbora Popelářová, Tibor Füzik, Tibor Botka, Martin Benešík, Pavol Bárdy, Roman Pantůček, Pavel Plevka

- Acquired movies (30,553): magnification 75,000 x, voltage 300 kV, pixel size 1.061 Å
- MotionCor2 and gCTF: micrograph alignment, CTF estimation, dose-weighting and micrograph selection (27,226 micrographs)
- Manual particle picking (23,947 virion capsids)
- Particle extraction: 400 px (4 x binned)
- Relion: 3D refinement (I4 symmetry)
- Symmetry expansion: I4 to C5 (12 x 23,947 capsid orientations)
- Relion: 2D classification with fixed orientations, mask on phage neck (21,731 particles aligned on capsid and neck)
- Neck sub-particle extraction (21,731 neck sub-particles, 1 x binned)

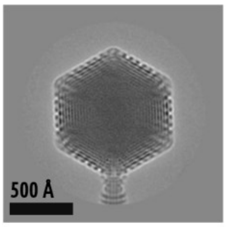

portal/neck 416 px
- 3D refinement in C6 symmetry: 4.2 Å res. ($FSC_{0.143}$)
- particles with best fit to C6 map kept: 16,293 particles
- local 3D refinement in C12 (EMD-18445, Fig. 5A): 4.2 Å res.

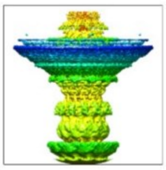

- model building of **portal** and **adaptor** into postprocessed map (1.08 Å/px, auto B-factor sharpening)

adaptor channel DNA
- C12 to C1 sym. expansion
- 3D classification in C1 w. fixed orientations, mask on adaptor chamber, 12 classes
- Reconstructed class (Fig. S23B): 9,050 unique particles

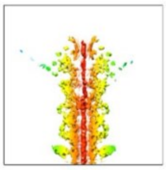

neck 480 px
- 3D refinement in C6 sym. (EMD-18462, Fig. 1B): 4.2 Å res.

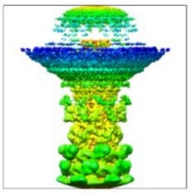

- model building of **stopper, tail terminator** and **neck decoration proteins** into postprocessed map (1.08 Å/px, auto B-factor sharpening)

neck/tail 480 px
- 3D refinement in C6 sym. (EMD-18912): 4.3 Å res.

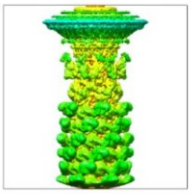

- model building of **tail sheath** and **tail tube** into postprocessed map (1.08 Å/px, auto B-factor sharpening)

tail 480 px
- 3D refinement in C6 sym.: 4.5 Å res.

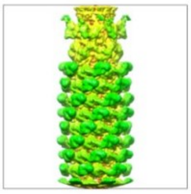

- C6 to C3 sym. expansion
- 3D classification in C3 sym. with fixed orientations, mask on tail channel, 2 classes
- Selected class: 13,126 unique particles
- local 3D refinement in C3 (EMD-18489): 6.6 Å res.

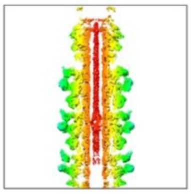

- fit of N-terminal model of **tail tape measure protein** into postprocessed map (1.08 Å/px, ΔB-factor = -50 Å^2)

tail channel DNA
- C3 to C1 sym. expansion
- 3D classification in C1 with fixed orientations, mask on tail channel, 3 classes
- Selected class: 9,102 unique particles
- local 3D refinement in C1 (EMD-18516, Fig. 7A): 8.6 Å res.

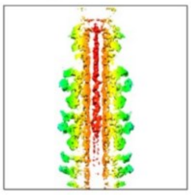

- fit of **B-form DNA** model into postprocessed map (1.08 Å/px, ΔB-factor = -50 Å^2)

Fig. S1. Cryo-EM reconstruction pathway of phage 812 virion. Flowchart of map reconstruction steps.

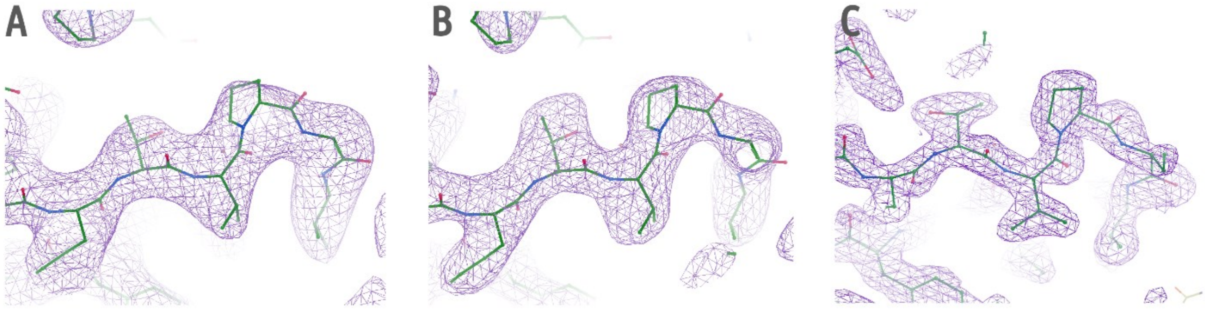

Fig. S2. Representative part of stopper protein maps. The maps are shown in mesh representation, with fitted molecular models depicted as sticks. **(A)** Cryo-EM reconstruction of phage 812 virion (EMD-18462, 4.2 Å resolution), **(B)** cryo-EM reconstruction of phage 812 genome-release intermediate (EMD-18048, 3.6 Å resolution), **(C)** electron density map determined by X-ray crystallography (PDB 8QGR, 2.2 Å resolution).

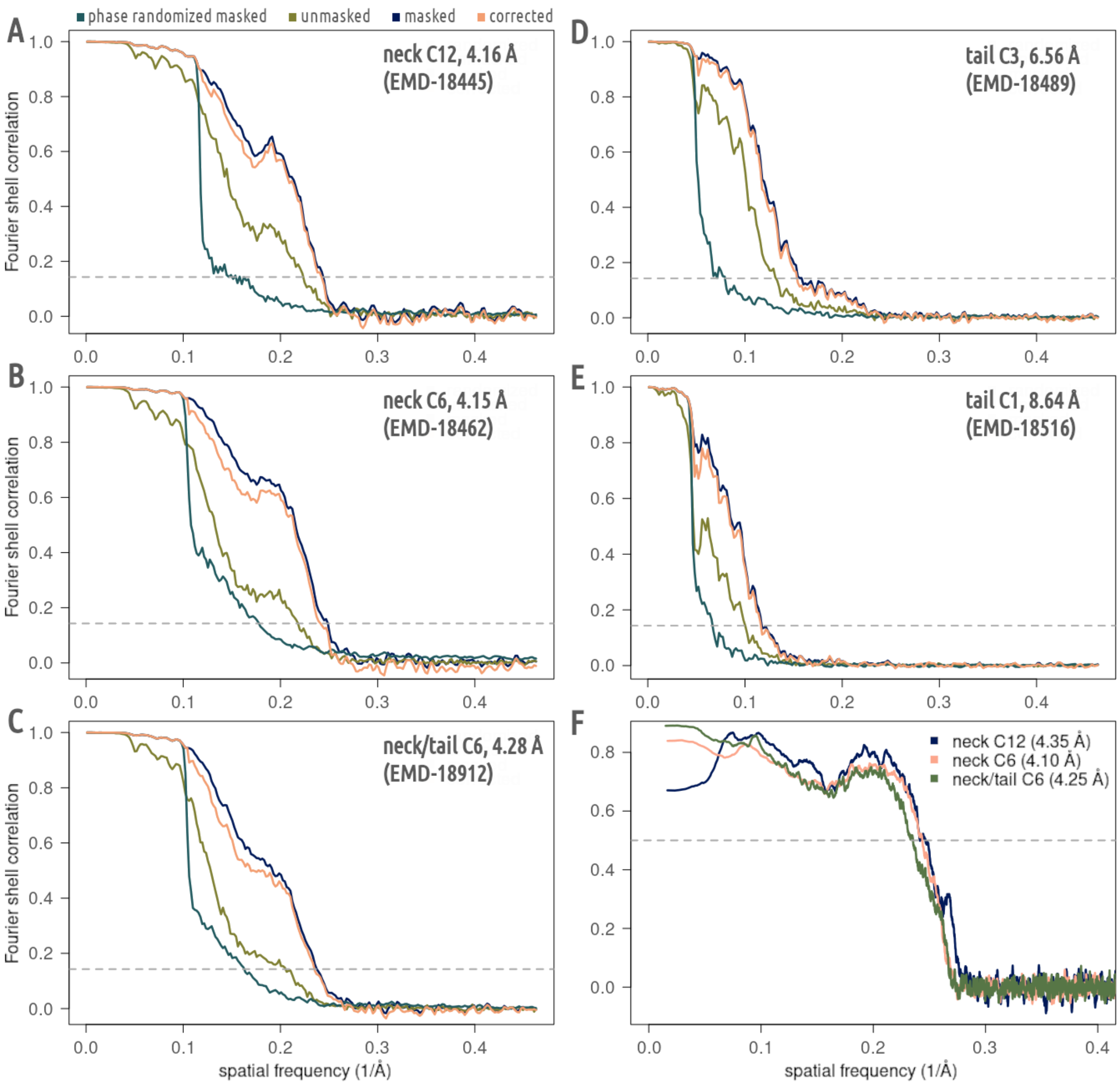

Fig. S3. Fourier shell correlation (FSC) plots for phage 812 virion. (A-E) FSC curves for gold-standard refinements deposited into EMDB (Table S1). Reported resolutions correspond to the 0.143 threshold criterion. **(F)** Masked FSC curves between refined maps and fitted models deposited into PDB. Reported resolutions correspond to the threshold 0.5.

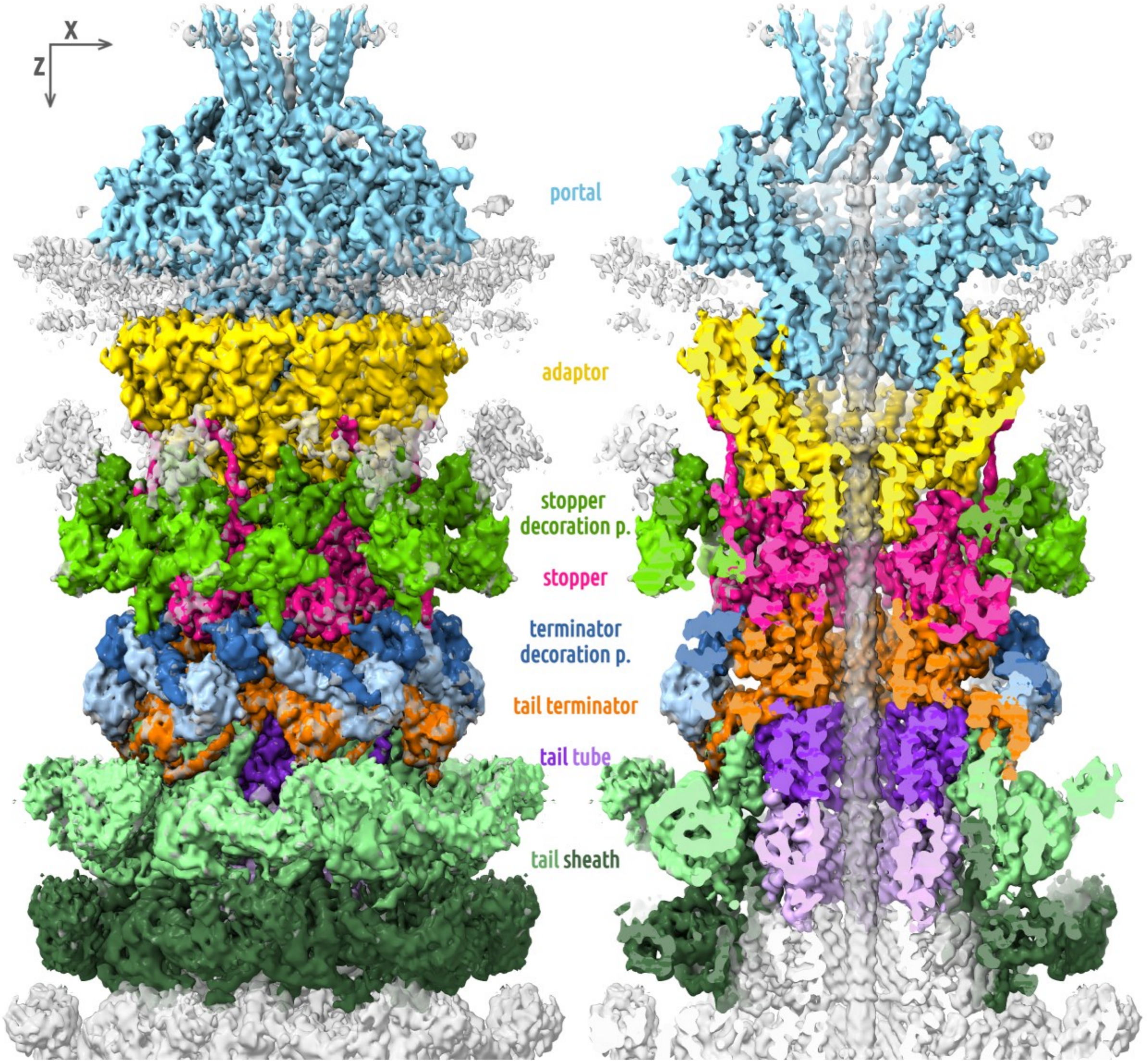

Fig. S4. Composite map of the neck of phage 812 virion. The unmasked map is shown as gray transparent surface. Map zones within a radius of 3 Å of fitted atomic models are colored according to the protein: portal in light blue, adaptor in gold, stopper in magenta, tail terminator in orange, stopper decoration in hues of forest green, terminator decoration in hues of steel blue, tail tube in hues of violet, and tail sheath in hues of desaturated green.

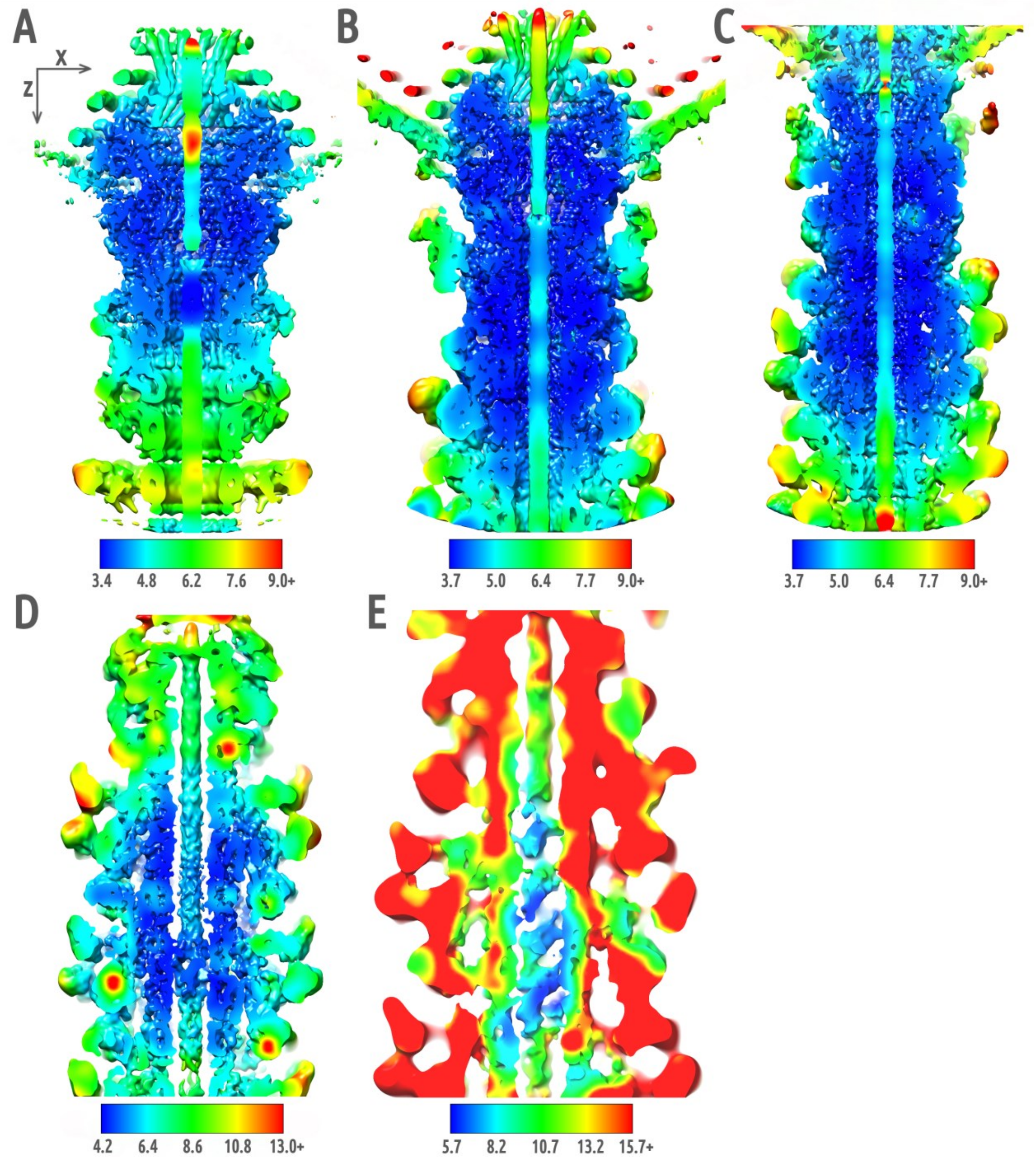

Fig. S5. Local resolution estimation for cryo-EM reconstructions of phage 812 virion. The maps are filtered and colored according to local resolution (in angstroms). **(A)** Neck solved with C12 symmetry (EMD-18445), **(B)** neck solved with C6 symmetry (EMD-18462), **(C)** neck/tail junction in C6 symmetry (EMD-18912), **(D)** neck/tail junction in C3 symmetry (EMD-18489), **(E)** neck/tail junction with DNA in the tail channel in C1 symmetry (EMD-18516).

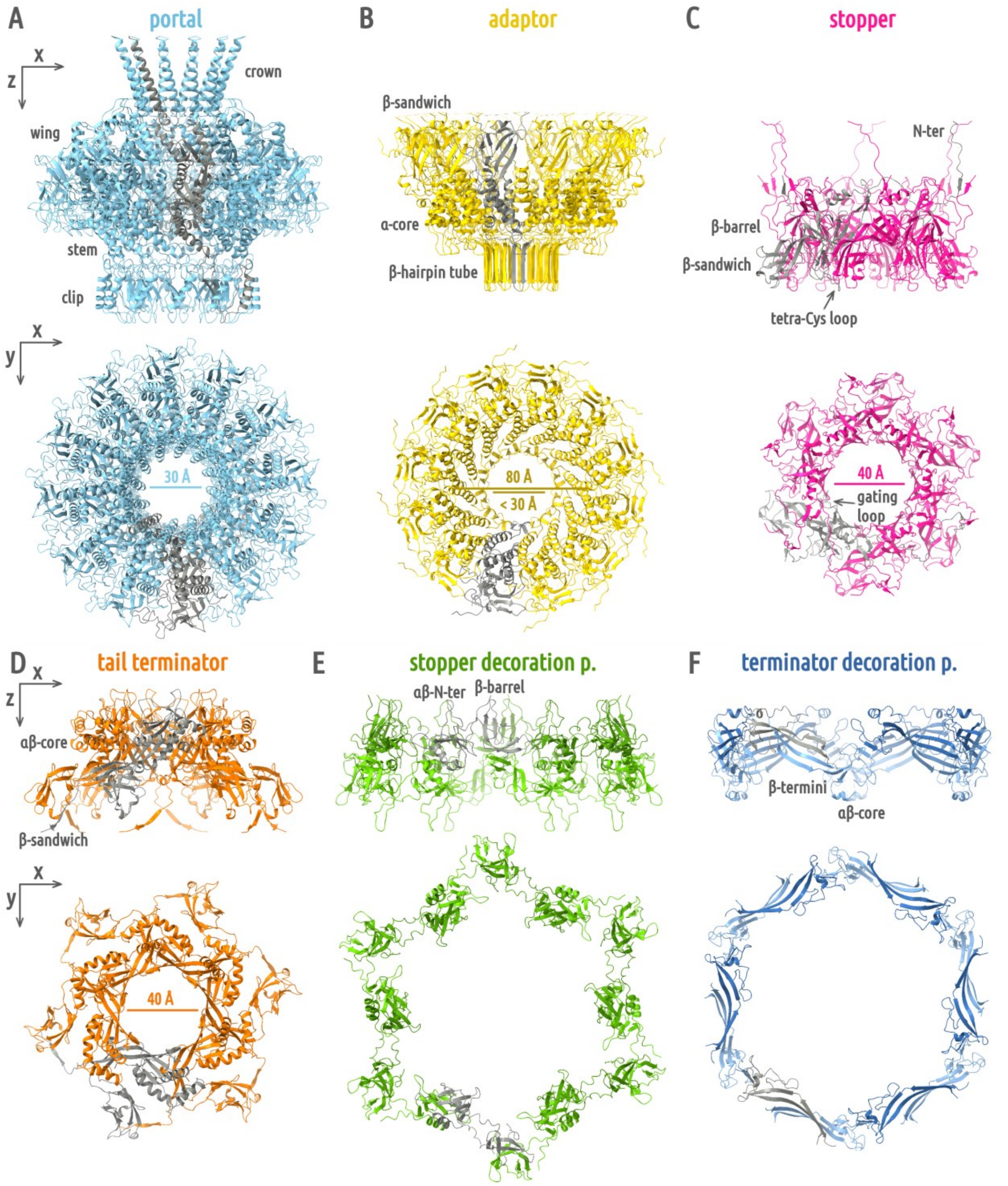

Fig. S6. Structures of protein complexes of phage 812 portal, neck, and tail (continues on next page). Cartoon representations of portal **(A)**, adaptor **(B)**, stopper **(C)**, tail terminator **(D)**, stopper decoration **(E)**, and terminator decoration **(F)** proteins from phage 812 virion. Head proximal tail sheath **(G)** and tail tube **(H)** proteins from phage 812 virion. Head-proximal tail sheath proteins **(I)** from phage 812 genome release intermediate with contracted tail (domains III and IV are not shown). One monomer per complex is shown in gray.

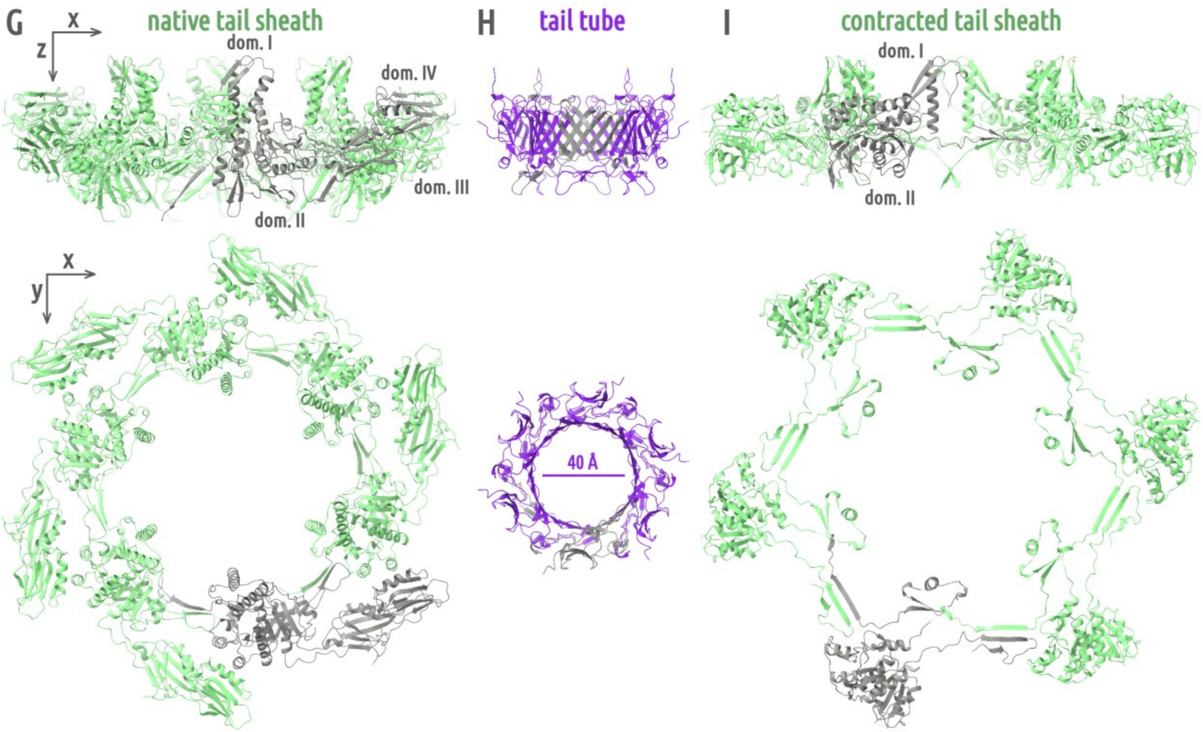

Fig. S6 (cont.). Structures of protein complexes of phage 812 portal, neck, and tail.

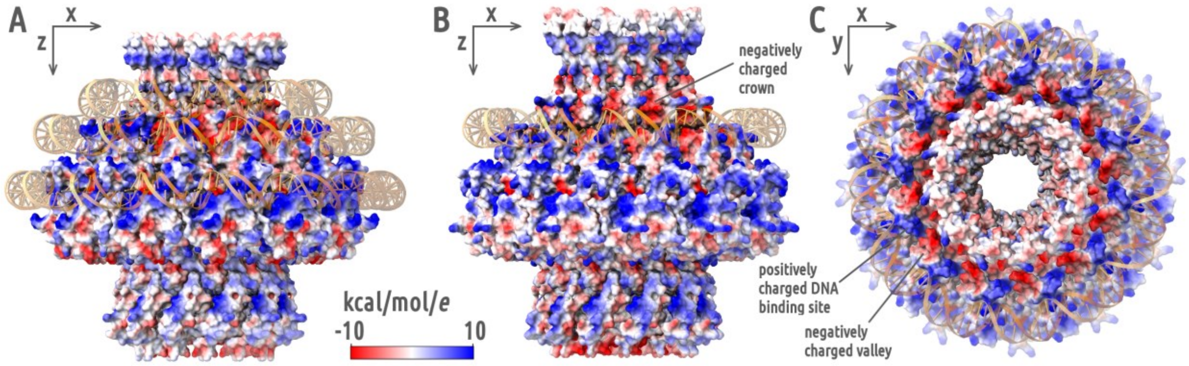

Fig. S7. Distribution of electrostatic potential on outer surface of portal dodecamers. Portal complexes are shown in surface representation for phage 812 virion **(A)** and genome release intermediate **(B and C)**. dsDNA chains modelled into the neighboring ring densities in C12-symmetrized maps are shown as beige cartoons at 30% transparency.

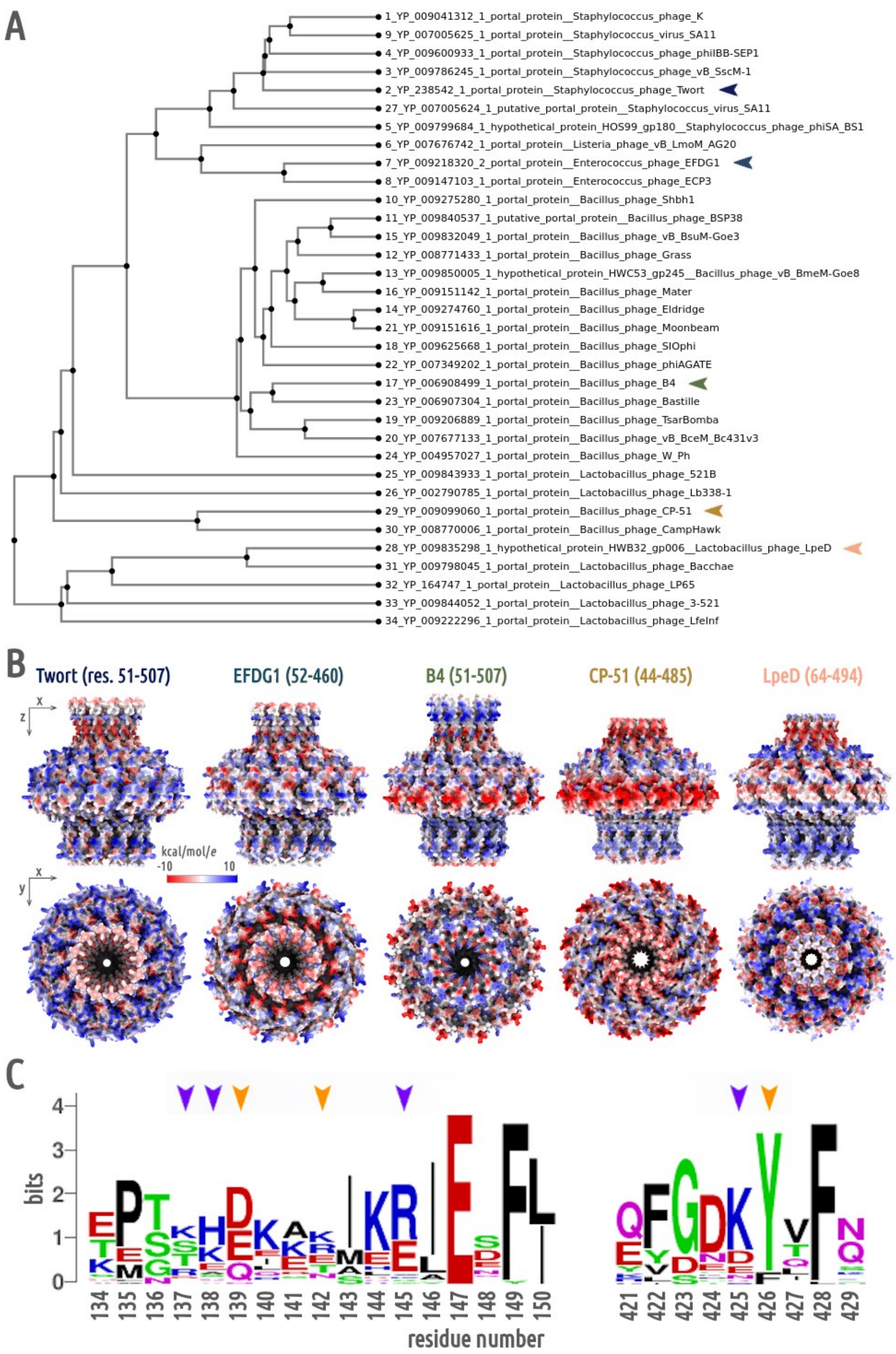

Fig. S8. Conservation of charged DNA binding site on wing domains of portal proteins. (A) Guide tree based on a multiple sequence alignment of portals from 33 representative species of *Herelleviridae* phages. *Staphylococcus* phage K (top) is the closest relative of phage 812, with a 100% sequence identity between their portal proteins. Bibliographic information about the phage species is collated in Table S10. **(B)** Electrostatic surface potential of modelled portal dodecamers for five phage species, marked with arrows in panel A. **(C)** Sequence conservation of the anchor DNA binding site in the 33 *Herelleviridae* species. Positively charged residues interacting with DNA in phage 812 are marked with purple arrowheads, those participating in the hydrogen bond network are marked with orange arrowheads.

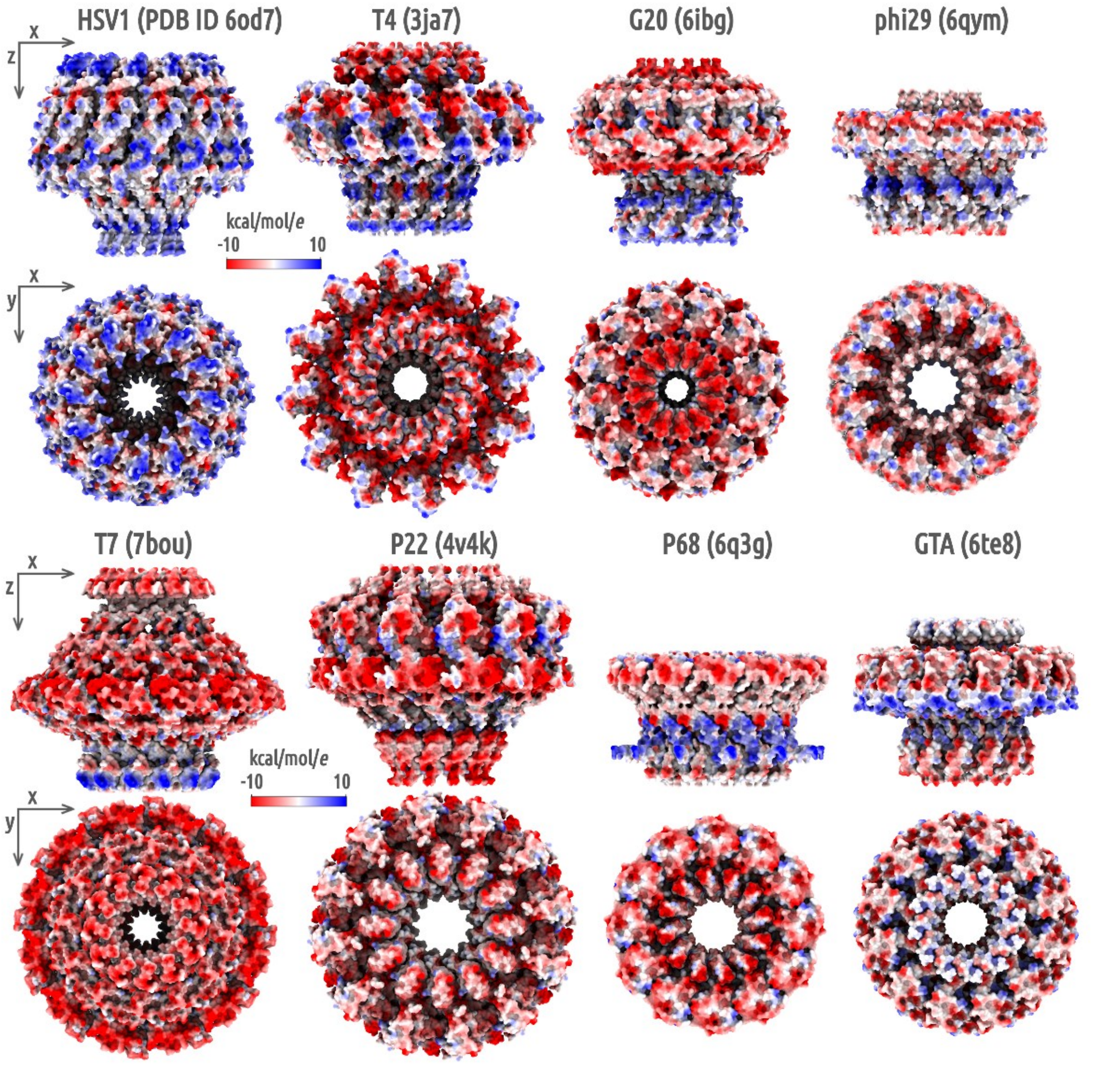

Fig. S9. Electrostatic surface potential of portal complexes. Portal proteins with known structures (retrieval date 2022-03-01, one example per taxonomic family selected) are shown in surface representation. Bibliographic information about reported portals is summarized in Table S11.

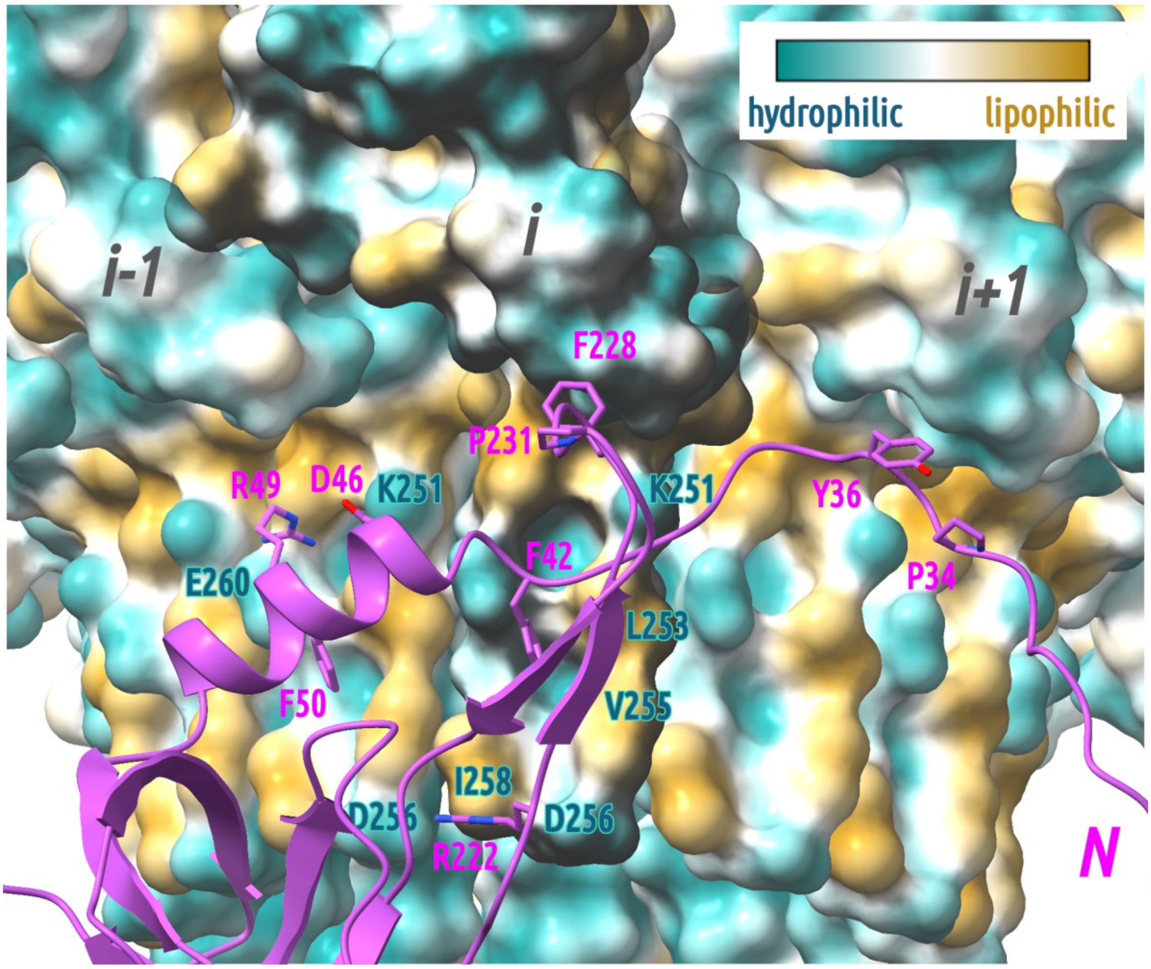

Fig. S10. Reduction of symmetry from dodecamer of adaptor proteins to hexamer of stopper proteins. The adaptor dodecamer is shown in surface representation colored by lipophilicity, and one monomer (labeled as *i*) is darkened for emphasis. One stopper protein is shown in magenta in cartoon representation. One stopper protein interacts on average with two adaptor proteins by replicating analogous intermolecular contacts: stopper Phe42 *vs.* Phe50 (to adaptor *i* and *i-1* Leu253, Val255 and Ile258); and Pro34-Tyr36 loop *vs.* Phe228-Pro231 loop (to adaptor *i* and *i+1* Pro64-Gly67 loops). The largest interface area is 661.1 Å^2 with adaptor *i*, and the second largest 423.2 Å^2 with *i-1*, which is further reinforced by salt bridges: from stopper Asp46 to adaptor *i-1* Lys251, stopper Arg49 to adaptor *i-1* Glu260, and stopper Arg222 to adaptor *i-1* Asp256. Finally, a 40-residues-long extended N-terminus of the stopper protein embraces the adaptor ring, bringing a single stopper protein in contact with five adaptor proteins.

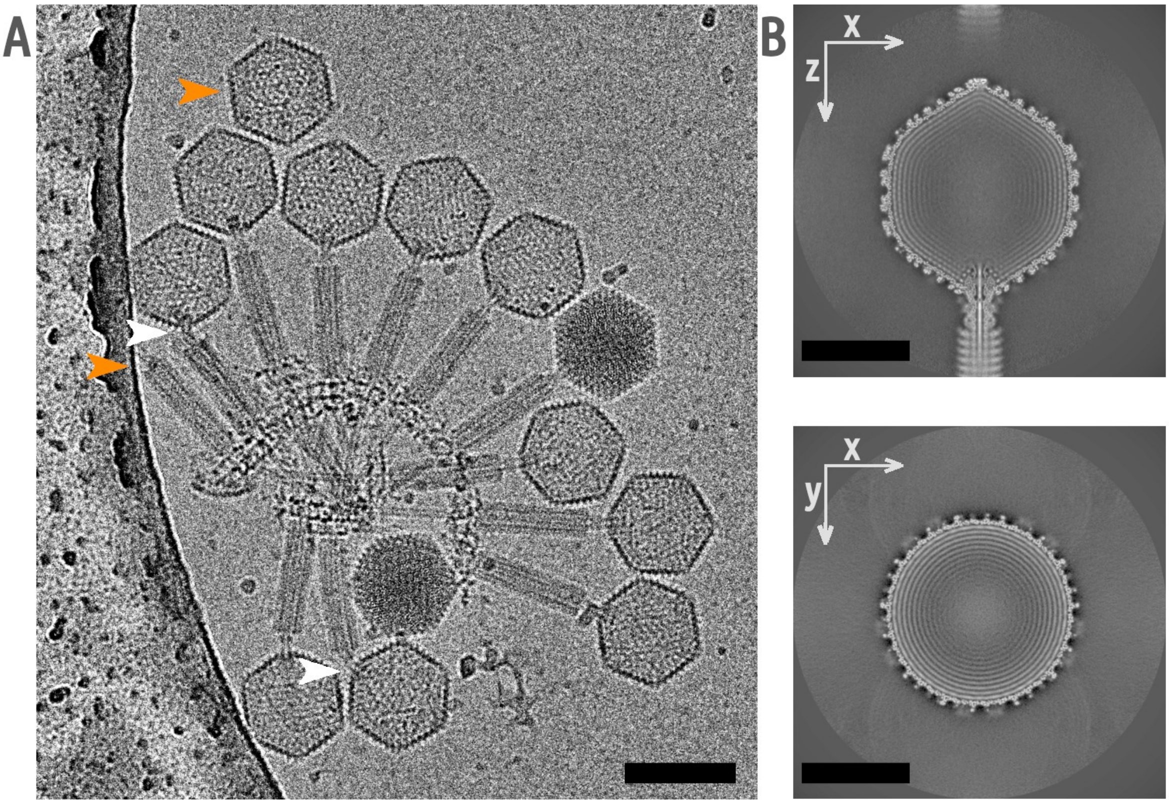

Fig. S11. Weakening of phage 812 neck induced by i*n vitro* contraction; organization of DNA in phage 812 heads. (A) Phage 812 particles lacking neck decoration proteins after tail contraction treatment. Particles bent at the neck are indicated with white arrowheads; particles broken at the neck with orange arrowheads. The scale corresponds to 1,000 Å. **(B)** Genome organization in phage 812 virion. Central XZ and XY slices through the virion reconstructed in C5 symmetry suggest that DNA spooling is coaxial to the neck-tail *z* axis. The scale corresponds to 500 Å.

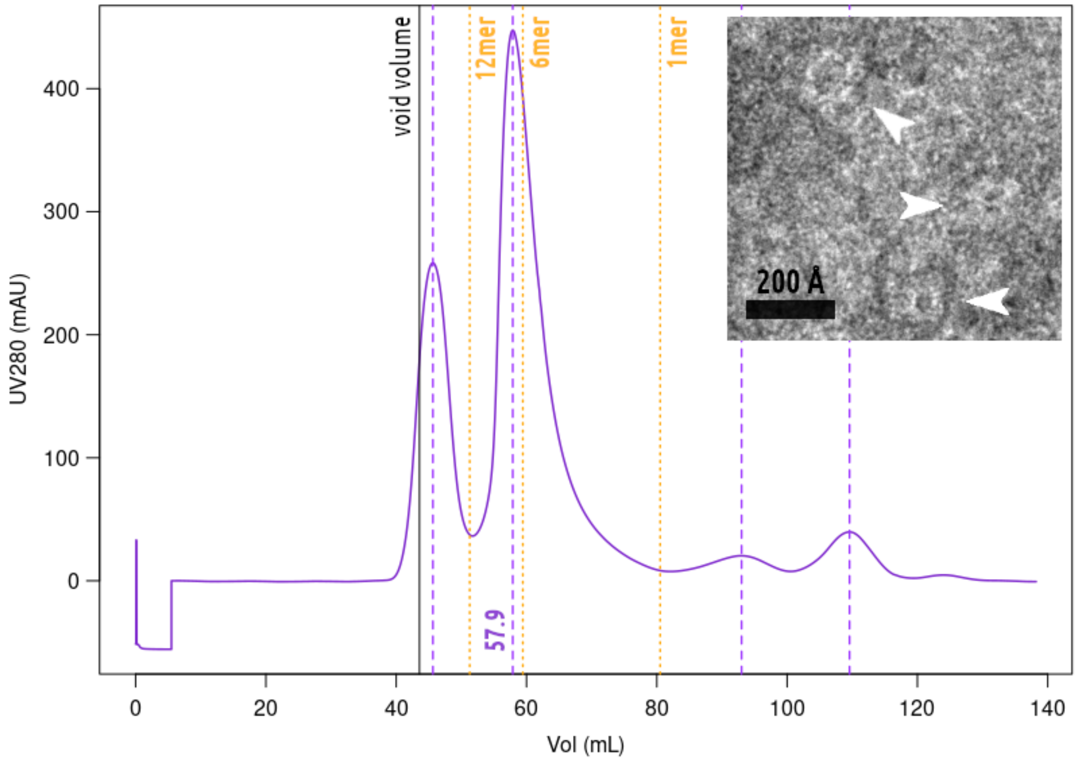

Fig. S12. Size exclusion chromatography profile of stopper protein gp97. The peak eluted at volume 57.9 mL was used for crystallization. The expected elution volumes of gp97 monomer, hexamer and dodecamer are marked in gold. The inset shows a cryo-EM image of the collected stopper protein sample.

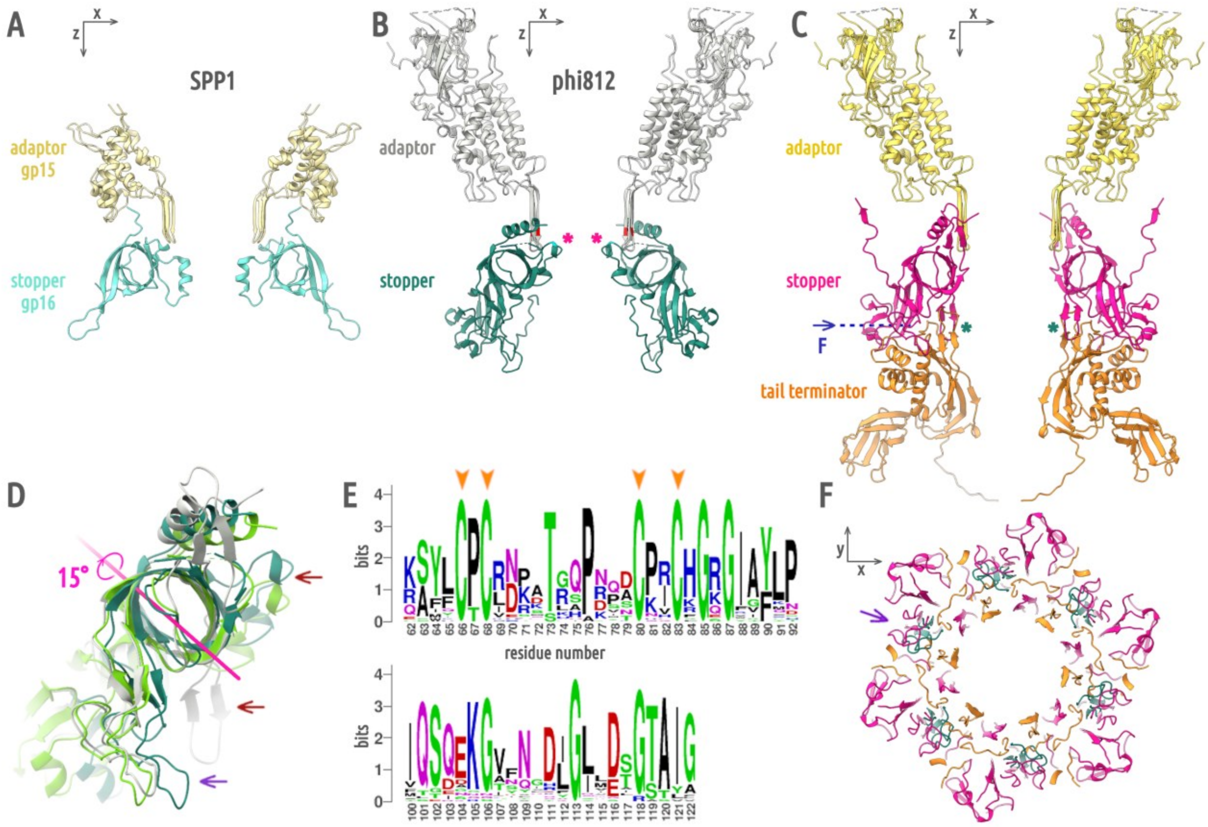

Fig. S13. Stopper complex in phage particle before and after tail attachment. (A) Structure of the adaptor (light gold) and stopper (light teal) complex in the tailless particle of phage SPP1[1]. Two stopper and four adaptor monomers are shown in cartoon representation. The stopper proteins of phages SPP1 and 812 have 9% sequence identity, and an RMSD of 6.1 Å over 42 matched Cα atoms belonging to the core β-barrel and the gating loop. **(B)** Model of the interaction of the stopper complex in its closed, crystal conformation (teal) with the adaptor complex (gray) in phage 812. Two stopper and four adaptor monomers are shown in cartoon representation. The β-hairpins of the adaptor tube insert into the bend of the closed stopper gating loop (marked with magenta asterisks), with putative favorable contacts between the Glu254 of adaptor proteins (red) and Lys105 of stopper proteins (cyan). **(C)** Interaction of the open stopper complex (magenta) in the virion with adaptor (gold) and tail terminator (orange) complexes. Extended N-termini of the stopper proteins (residues 1-39) are not shown for clarity. The gating loop of the stopper protein in the open conformation is sandwiched between two loops (residues 60-73 and 236-243) of the tail terminator protein (teal asterisks). **(D)** Rearrangements within a stopper monomer. The closed hexameric stopper complex (teal) was aligned to the open complex (gray) by minimizing the pairwise backbone RMSD of the six core domains (β-barrels and tetra-cysteine loops), then a monomer in the closed conformation was realigned (light green) to the β-barrel of the open conformation to illustrate the concerted rotation of the β-barrel together with the tetra-cysteine loop (purple arrow) during stopper opening, which leads to the displacement of the gating loop (red arrows) from the adaptor β-hairpin tube. The rotation axis is shown in magenta, extended protein termini are omitted for clarity. **(E)** Sequence conservation of the tetra-cysteine motif (top) and the gating loop (bottom) in stopper proteins from the *Herelleviridae* family (see Table S10 for bibliographic information). The four cysteines are marked with orange arrowheads. **(F)** Cross-section through the neck channel of phage 812 at the interface between stopper and tail terminator complexes (marked with a navy arrow in panel C). Binding of tail terminator complex (orange) displaces the tetra-cysteine loops (one marked with a purple arrow) of the stopper complex outwards (closed conformation in teal, open in magenta).

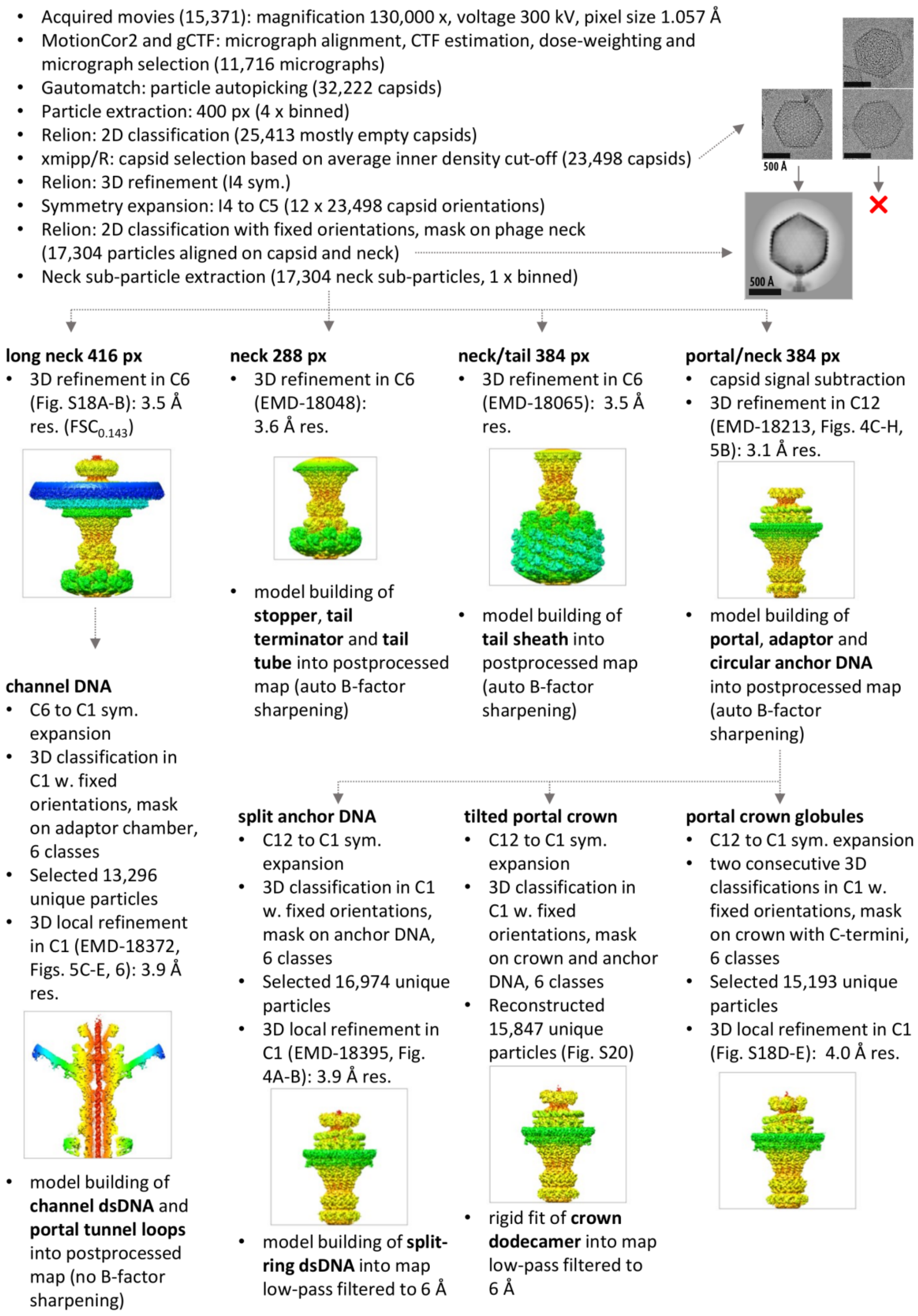

Fig. S14. Cryo-EM reconstruction pathway of phage 812 genome release intermediate. Flowchart of map reconstruction steps.

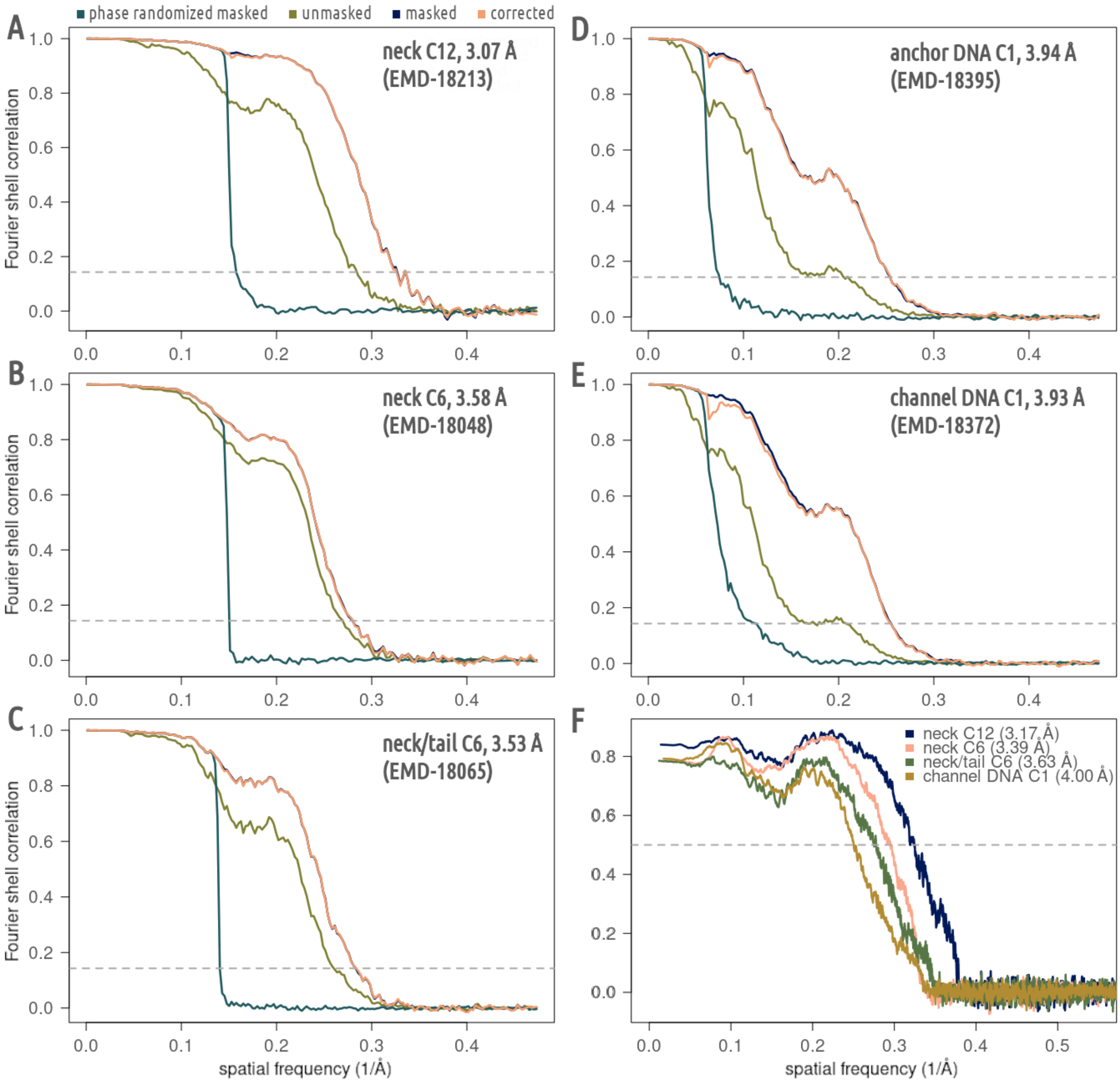

Fig. S15. Fourier shell correlation (FSC) plots for phage 812 genome release intermediate. (A-E) FSC curves for gold-standard refinements deposited into EMDB (Table S1). Reported resolutions correspond to the 0.143 threshold criterion. **(F)** Masked FSC curves between refined maps and fitted models deposited into PDB. Reported resolutions correspond to the threshold 0.5.

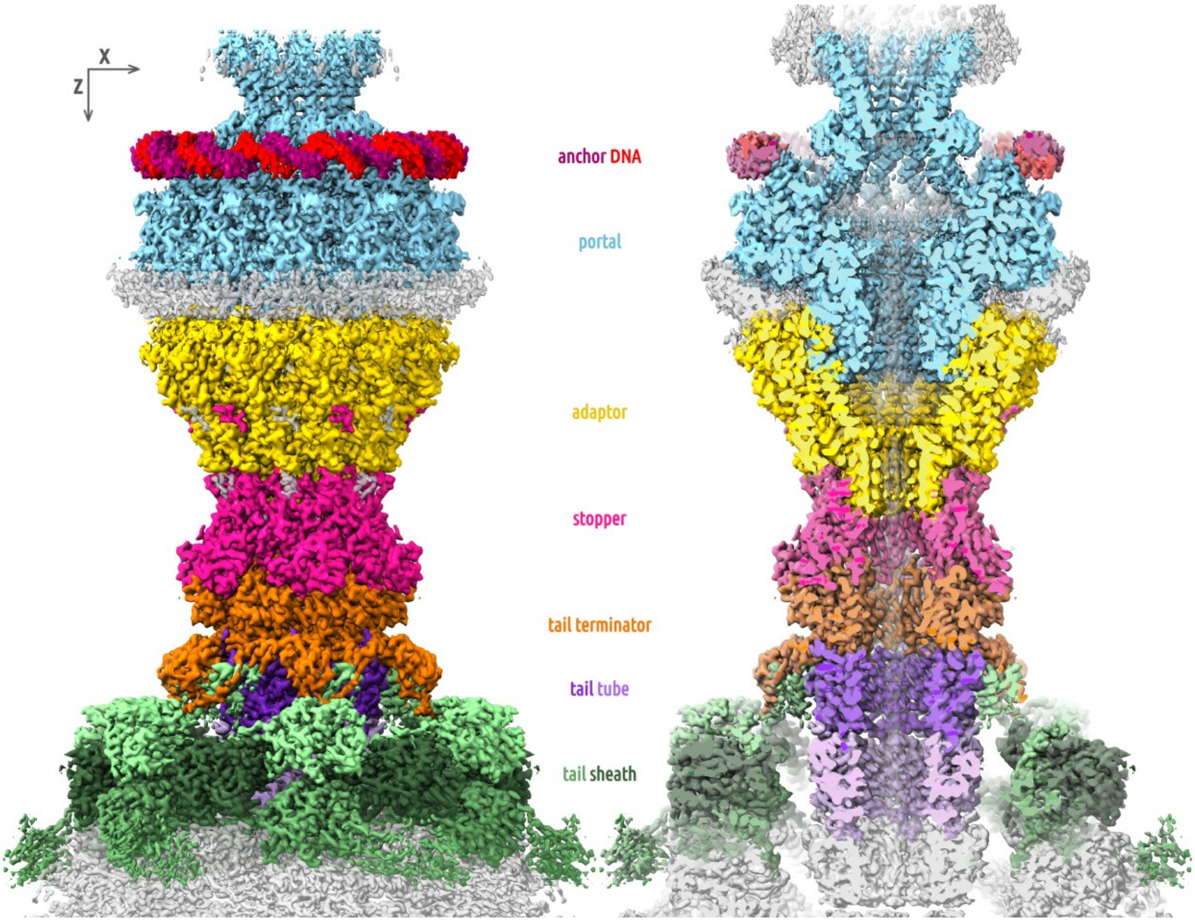

Fig. S16. Composite map of the neck of phage 812 genome release intermediate. The unmasked map is shown as gray transparent surface. Map zones within a radius of 3 Å of fitted atomic models are colored according to the model: anchor DNA in hues of red, portal in light blue, adaptor in gold, stopper in magenta, tail terminator in orange, tail tube in hues of violet, and tail sheath in hues of desaturated green.

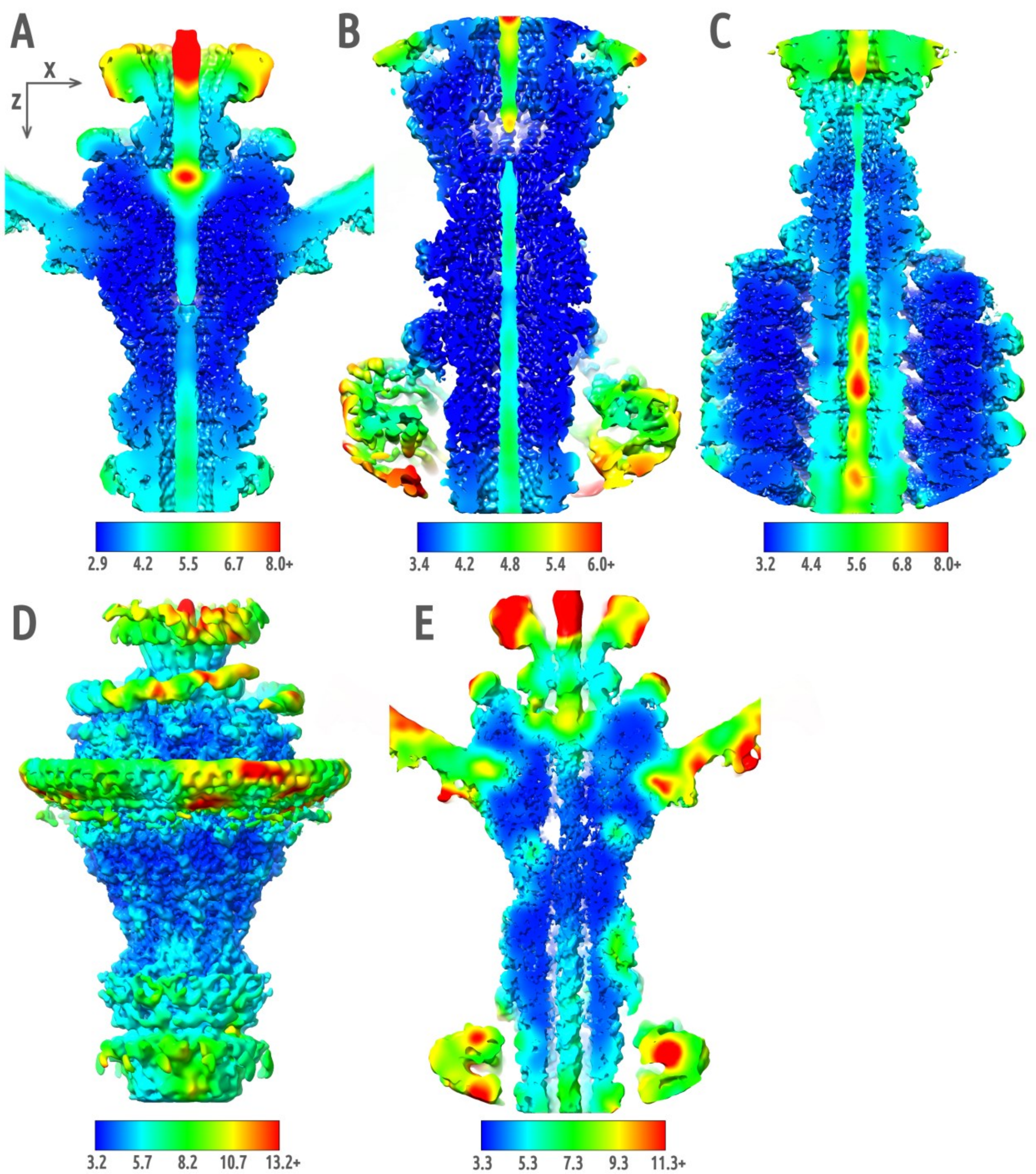

Fig. S17. Local resolution estimation for cryo-EM reconstructions of phage 812 genome release intermediate. The maps are filtered and colored according to local resolution (in angstroms). **(A)** Neck solved with C12 symmetry (EMD-18213), **(B)** neck solved with C6 symmetry (EMD-18048), **(C)** neck/tail junction in C6 symmetry (EMD-18065), **(D)** anchor DNA and portal complex in C1 symmetry (EMD-18395), **(E)** DNA inside the neck channel in C1 symmetry (EMD-18372).

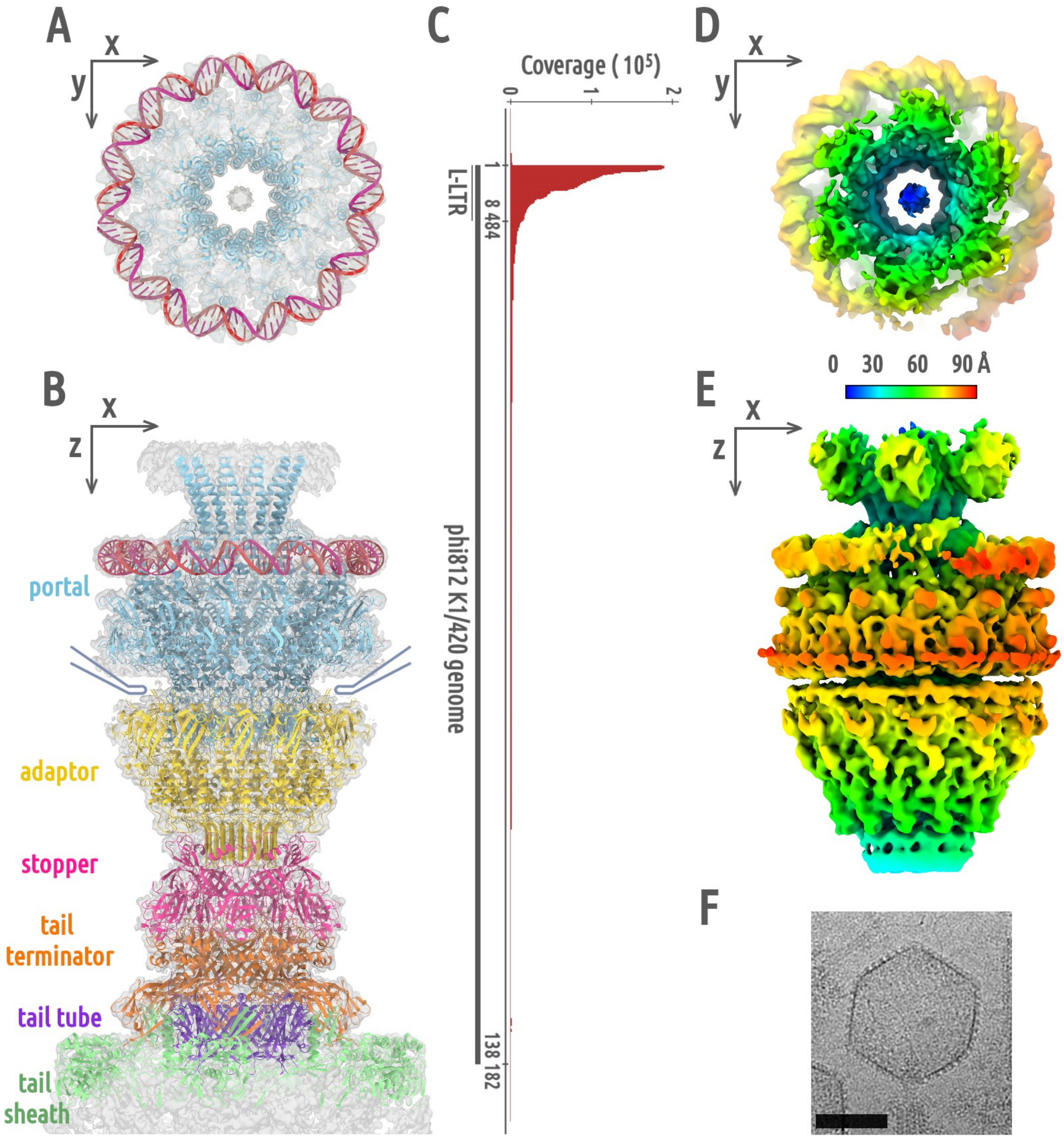

Fig. S18. Neck of phage 812 genome release intermediate. (A-B) C6-symmetrized cryo-EM map, shown in gray, is overlayed with the protein and DNA models in cartoon representation. The color coding of the proteins is identical to Fig. 1; the DNA is in red: primary red for the forward strand turning with a right-hand orientation along *z*, rose red for the reverse strand. **(C)** Mapping of sequence reads of DNA remaining in phage 812 capsids after genome release onto phage 812 genome. **(D-E)** Asymmetric reconstruction of the portal protein crown domains, low-pass filtered to 6Å, showing the twelve C-termini (residues 508-563) of portal proteins organized into six semi-folded globular structures. The map surface is colored according to the distance from the neck *z* axis. **(F)** Cryo-EM projection image of a genome-releasing phage capsid and neck with a loop of DNA anchored at the portal complex inside the capsid. The scale corresponds to 500 Å.

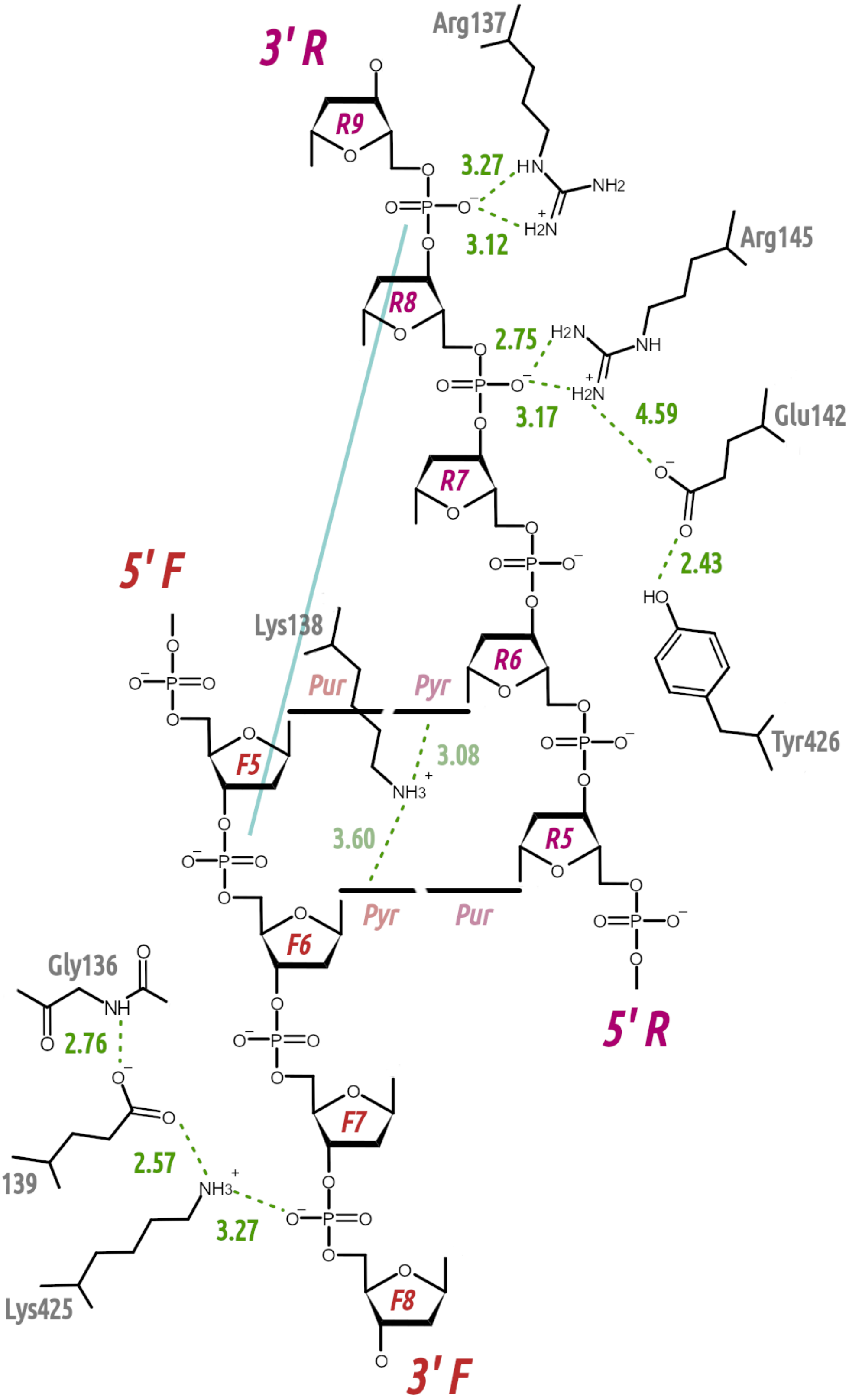

Fig. S19. Interface between portal protein and anchor DNA. Schematic representation of intermolecular contacts between one portal subunit and a section of the anchor DNA in the genome release intermediate of phage 812. The distances are indicated in angstroms and shown in green. The span of the minor groove at its narrowest point is indicated by a turquoise line.

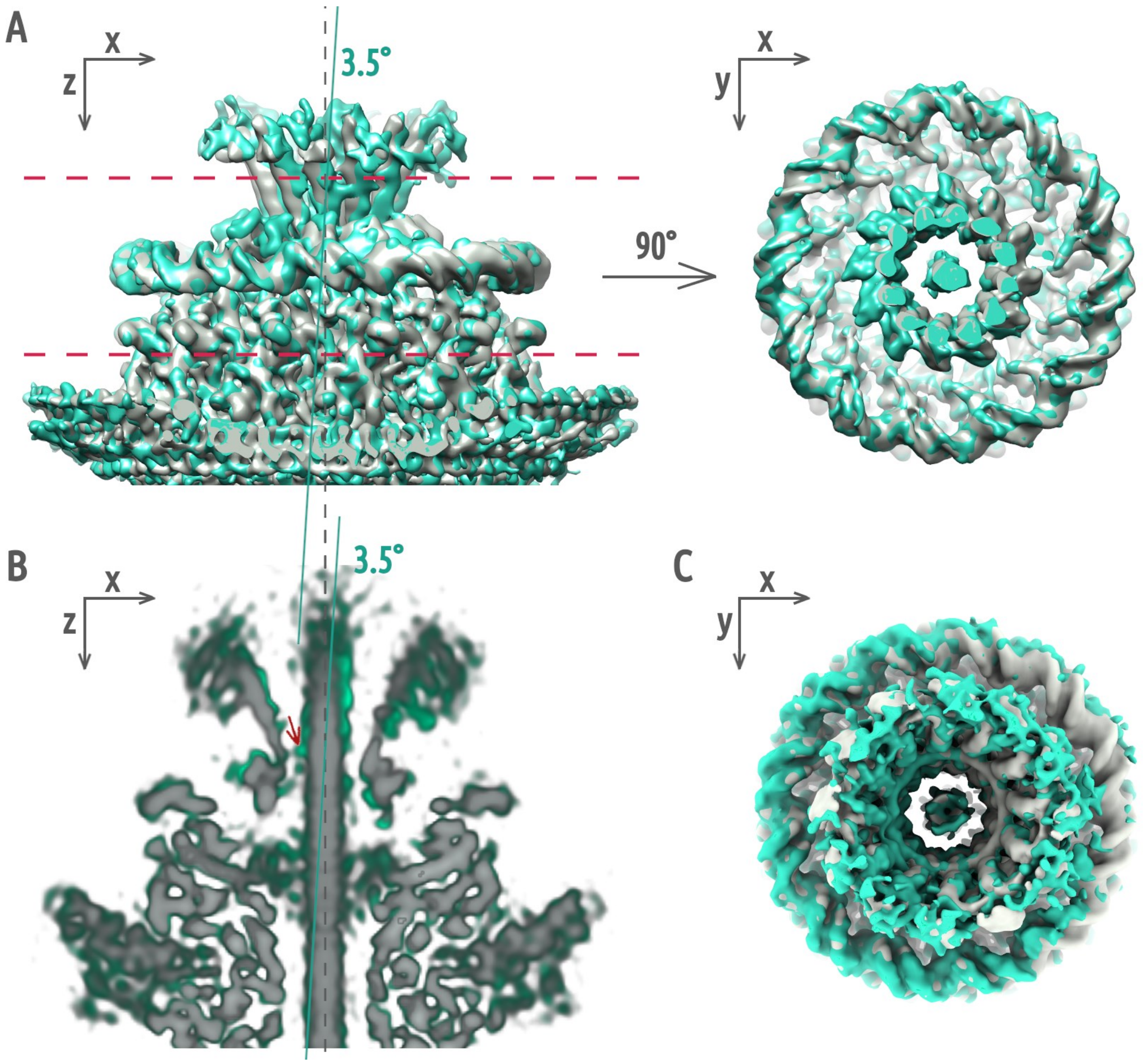

Fig. S20. Portal crown is tilted 3.5° in genome release intermediate. (A) Asymmetric cryo-EM density reconstructed with the focus on the portal crown domains is shown in teal green, the 12-fold symmetrized density is in gray. The central axis of the symmetrized density is denoted with a dashed gray line, the axis of the tilted crown density with a teal green line. The rotation axis of the tilt is oriented along -*y*. Dashed red lines indicate the position of clip planes in the rotated view on the left. **(B)** Overlay of central *xz* slices through the asymmetric (teal green) and symmetrized (gray) density. The reconstructions are identical to those shown in panel A. A red arrow indicates the bend in the channel DNA. **(C)** Asymmetric density reconstructed with the focus on crown domains of the portal proteins and the anchor DNA (teal green) overlayed with the C12-symmetrized reconstruction (gray) shows that the crown tilts towards the opening in the anchor DNA split ring. The rotation axis of the tilt is oriented along -*y*.

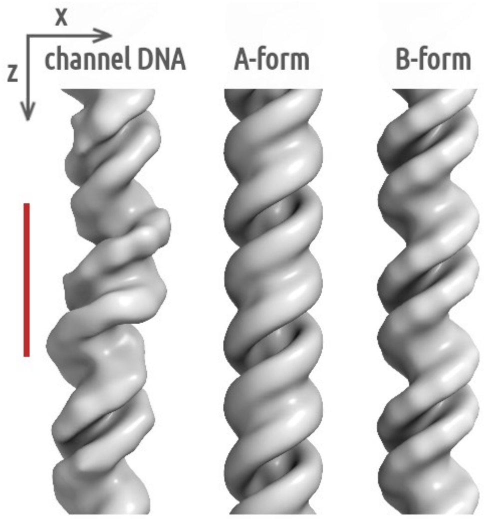

Fig. S21. Comparison of DNA structure inside neck channel of phage 812 genome release intermediate with ideal dsDNA. The asymmetric cryo-EM density inside the adaptor channel was zoned around DNA and low-pass filtered to 7 Å. The red segment indicates the position of the A-form DNA. The densities of A and B-form dsDNA were simulated from ideal atomic models and low-pass filtered to 7 Å.

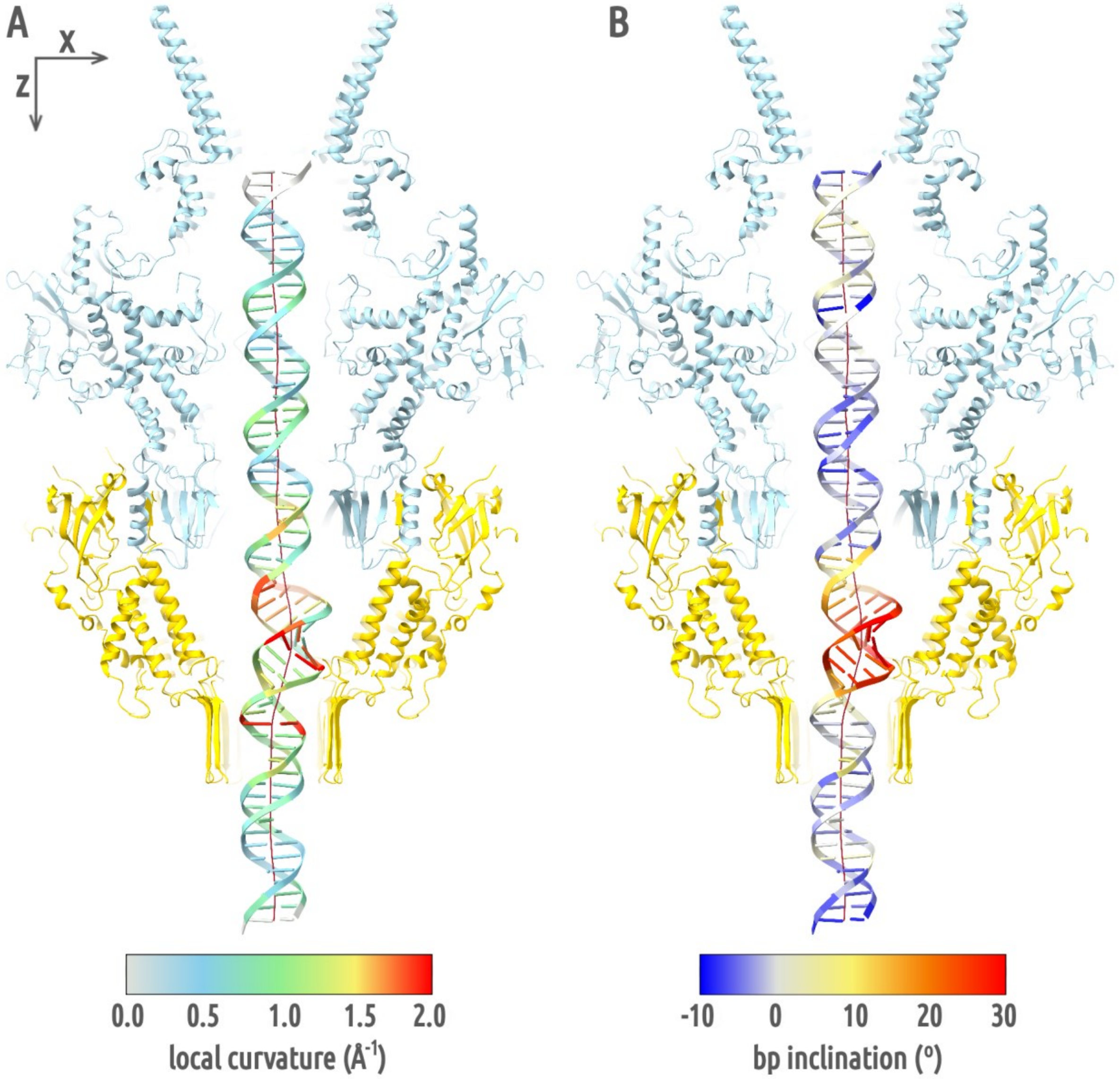

Fig. S22. Geometry of the channel DNA in the genome release intermediate. Portal (light blue) and adaptor (gold) complexes and the DNA are shown as cartoons. **(A)** DNA base pairs are colored according to local axial curvature. Prominent bending sites are located near the two B/A-form junctions and inside adaptor β-hairpin tube. **(B)** DNA base pairs are colored according to base pair inclination. Expected inclination angles are *ca* -2° for B-form DNA and 20° for A-form DNA.

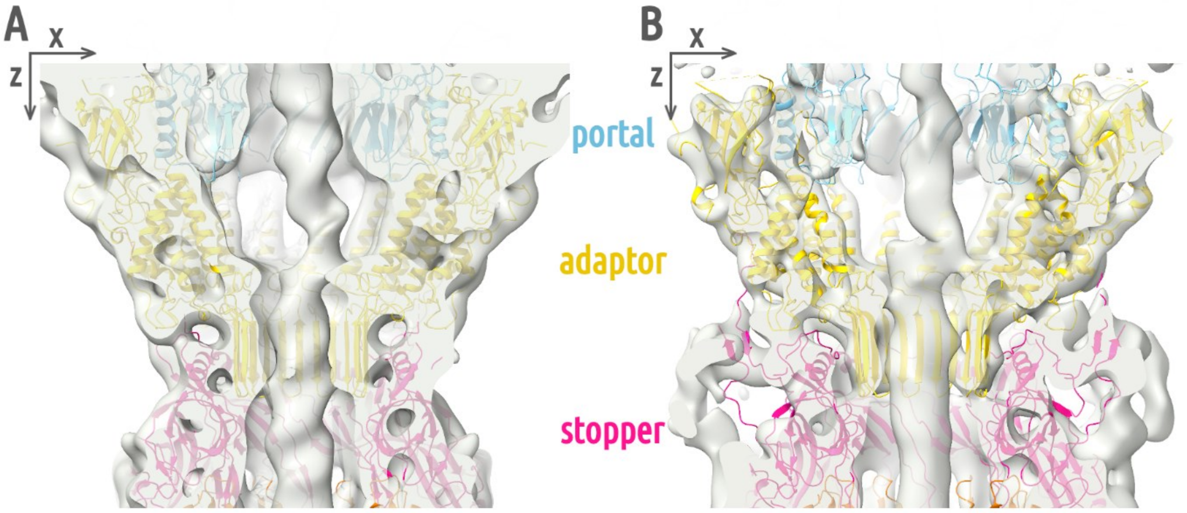

Fig. S23. Channel dsDNA changes to A conformation inside phage 812 adaptor chamber. Asymmetric reconstructions of the channel DNA density, low-pass filtered to 11 Å, in the genome release intermediate **(A)** and the virion **(B)**.

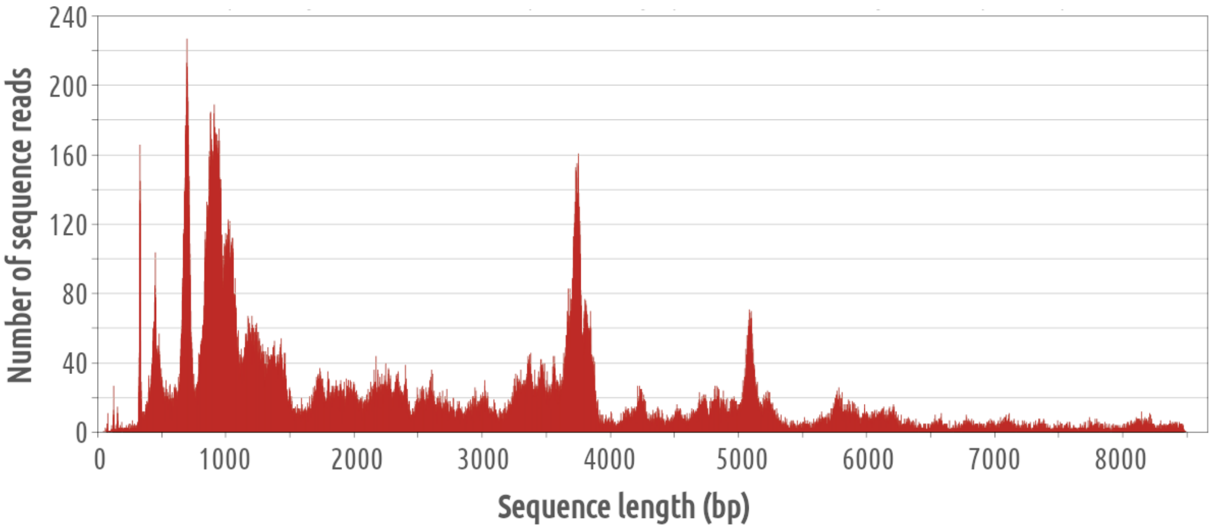

Fig. S24. Histogram of sequence lengths (bp) of DNA remaining in phage 812 capsids after genome ejection. Only trimmed reads starting exactly at the first nucleotide of the long terminal repeat are included.

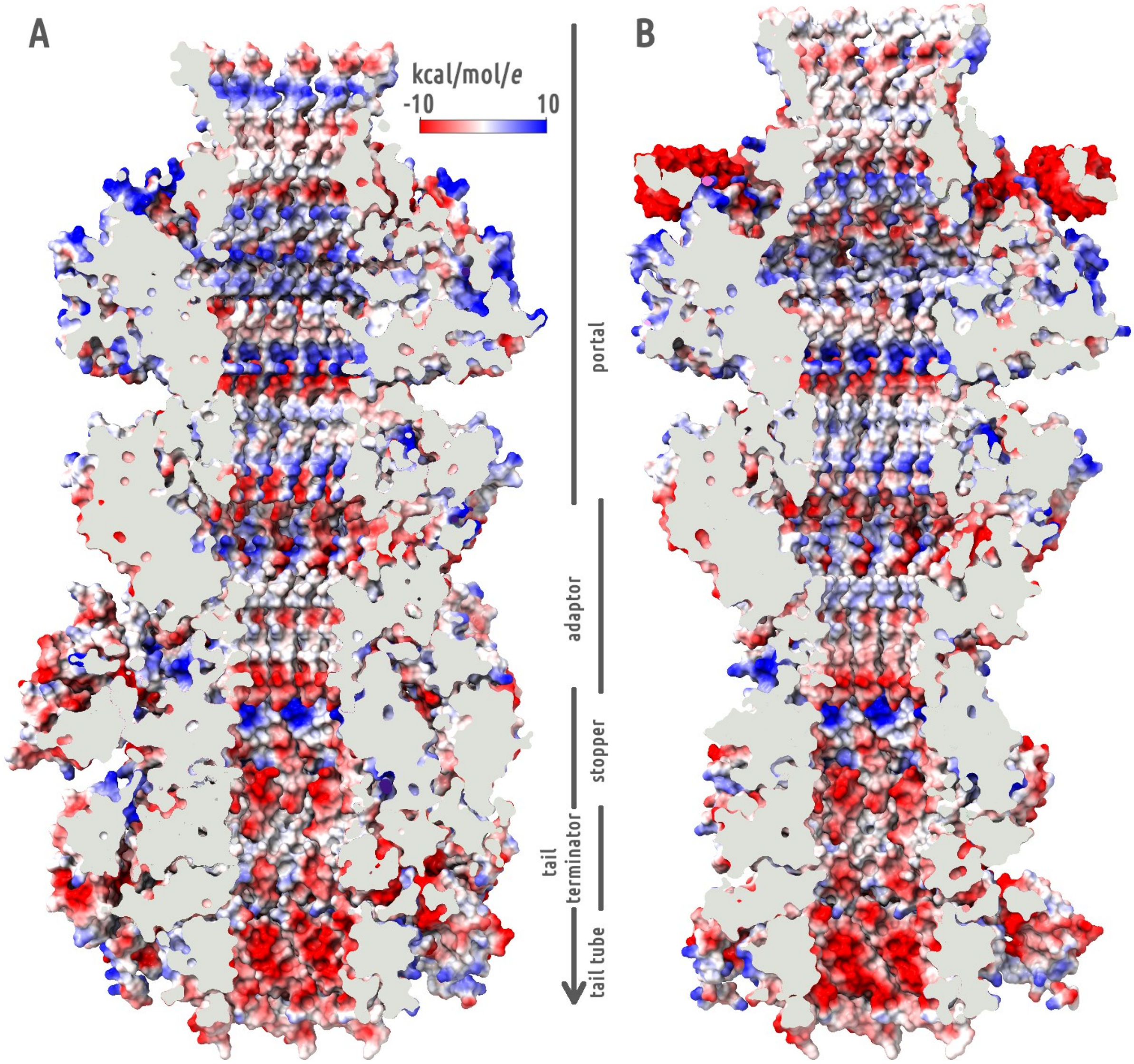

Fig. S25. Distribution of electrostatic surface potential inside phage 812 neck channel. Neck proteins of the virion **(A)** and the genome release intermediate **(B)** are shown in surface representation. The length of the channel formed by individual proteins is indicated by annotated segments. Disordered portal tunnel loops were not built and are missing from the visualization.

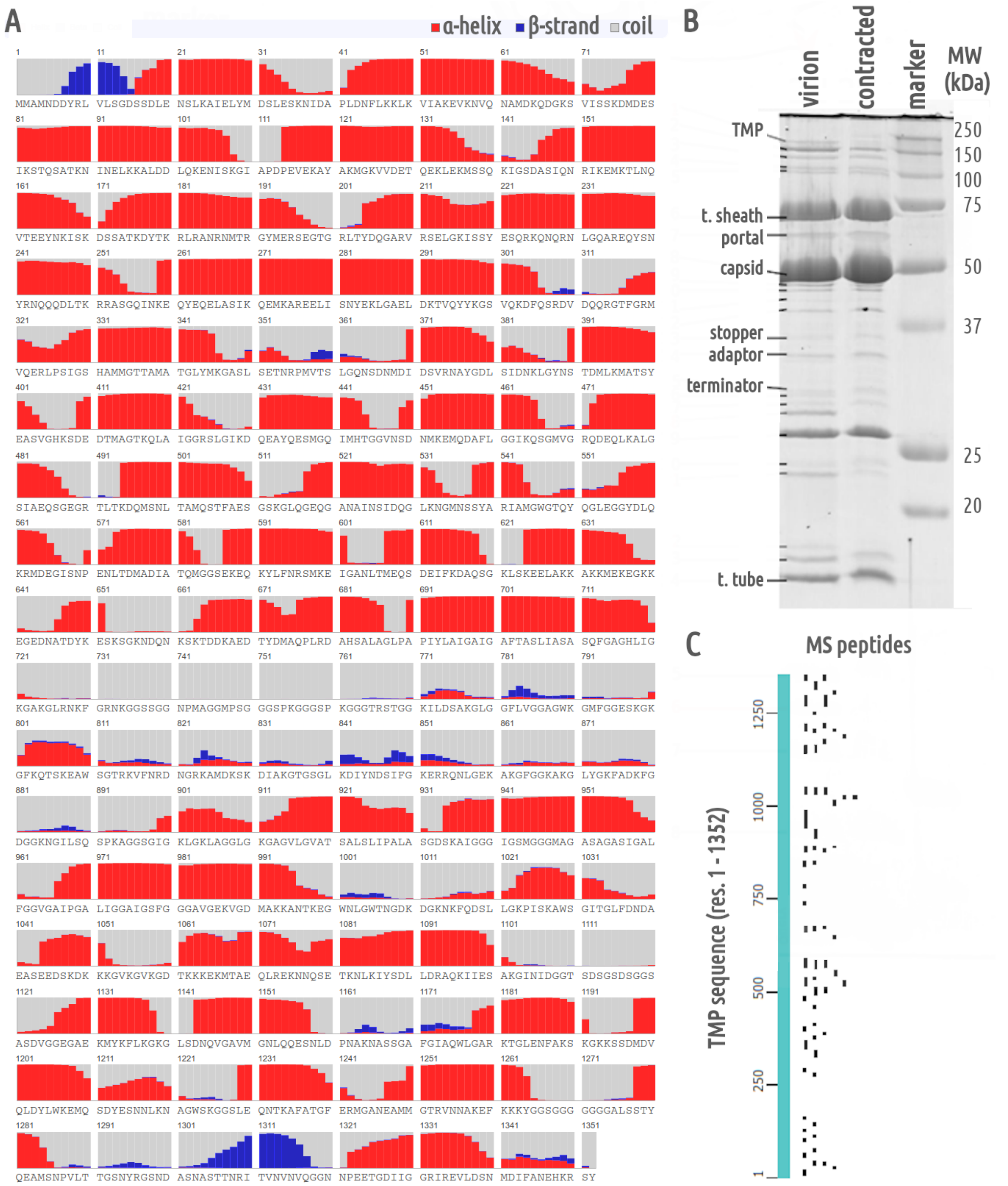

Fig. S26. Tail tape measure protein (TMP). (A) Secondary structure prediction of phage 812 TMP gp109. **(B)** SDS-PAGE of phage 812 virions and genome release intermediates after tail contraction. TMP is the major component of the first band. The unedited gel image is shown in Fig. S27. **(C)** Alignment of TMP mass spectrometry peptides with a statistically significant score ($p < 0.05$) to the primary sequence of TMP gp109.

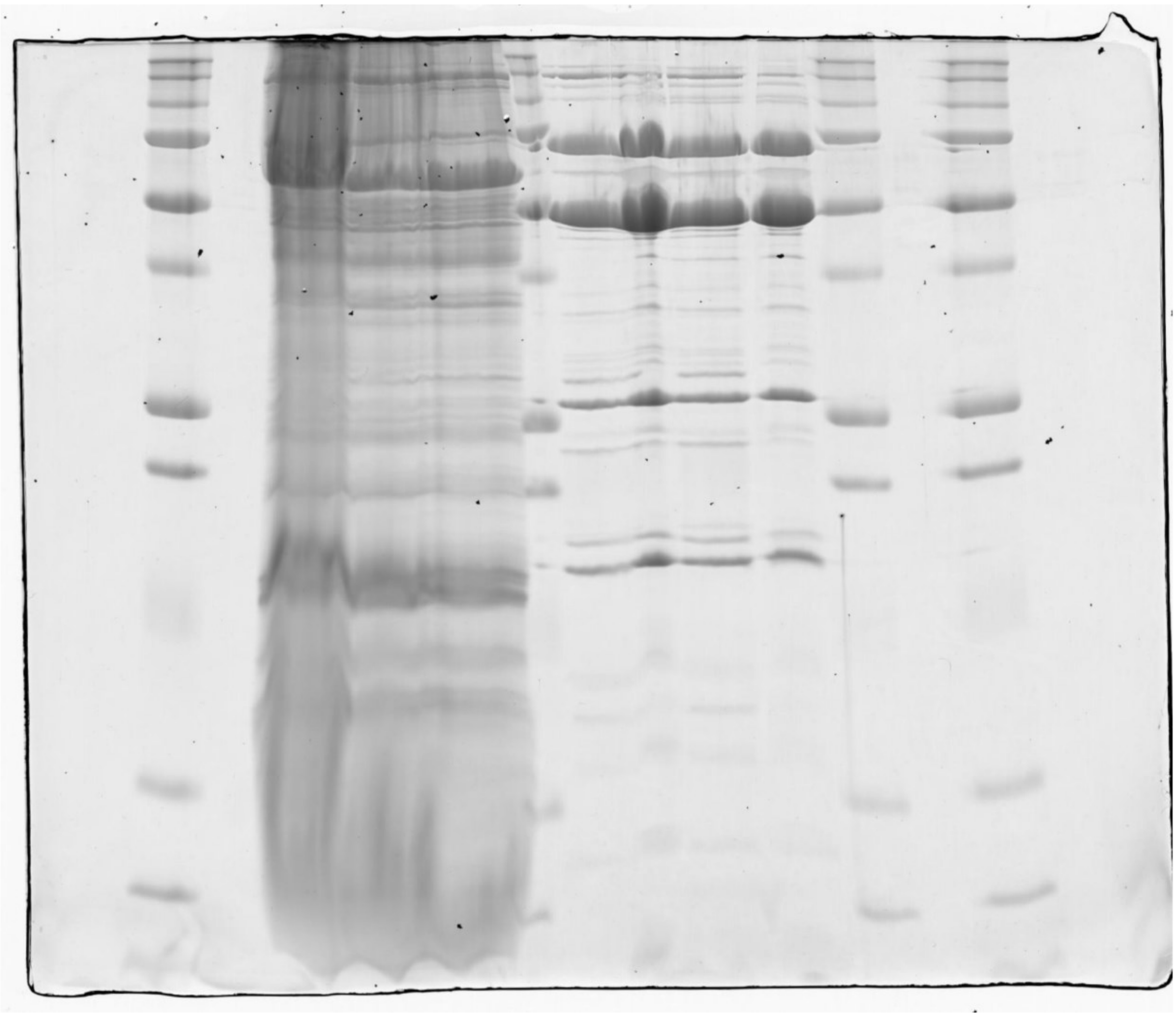

Fig. S27. Unedited image of SDS-PAGE described in Fig. S26B.

Table S1. Cryo-EM data acquisition, processing, and validation statistics for phage 812 particles.

	phage 812 virion					phage 812 genome release intermediate				
	**neck C12**	**neck C6**	**neck/tail C6**	**neck/tail C3**	**neck/tail C1**	**neck C12**	**anchor DNA**	**channel DNA**	**neck C6**	**neck/tail C6**
**Data acquisition**										
Magnification	75,000	75,000	75,000	75,000	75,000	130,000	130,000	130,000	130,000	130,000
Voltage (kV)	300	300	300	300	300	300	300	300	300	300
No. of frames	16	16	16	16	16	40	40	40	40	40
Electron exposure ($e^-/Å^2$)	49	49	49	49	49	42	42	42	42	42
Underfocus range (μm)	1.2 – 2.4	1.2 – 2.4	1.2 – 2.4	1.2 – 2.4	1.2 – 2.4	1.0 – 2.4	1.0 – 2.4	1.0 – 2.4	1.0 – 2.4	1.0 – 2.4
**Data processing**										
Initial no. of particles	21,731	21,731	21,731	21,731	21,731	17,304	17,304	17,304	17,304	17,304
Initial no. of particles	16,293	21,731	21,731	13,126	9,102	17,304	16,974	13,296	17,304	17,304
Symmetry	C12	C6	C6	C3	C1	C12	C1	C1	C6	C6
Pixel size (Å/px)	1.080	1.080	1.080	1.080	1.080	1.057	1.057	1.057	1.057	1.057
Map resolution ($FSC_{0.143}$) (Å)	4.16	4.15	4.28	6.56	8.64	3.07	3.94	3.93	3.58	3.53
Map resolution range (Å)	3.1 – 22.7	3.5 – 11.1	3.5 – 16.9	4.2 – 24.7	5.6 – 50.0	2.7 – 10.2	3.1 – 26.2	3.3 – 24.4	3.0 – 8.3	3.0 – 11.9
Map sharpening B factor ($Å^2$)	-179.3	-139.8	-134.0	-50.0	-50.0	-74.5	0.0	0.0	-110.4	-97.0
**EMDB entry ID**	EMD-18445	EMD-18462	EMD-18912	EMD-18489	EMD-18516	EMD-18213	EMD-18395	EMD-18372	EMD-18048	EMD-18065
**Atomic model**										
Initial model	predicted	predicted	predicted	-	-	predicted	-	predicted	predicted	predicted
Model resolution ($FSC_{0.5}$) (Å)	4.35	4.10	4.25	-	-	3.17	-	4.0	3.39	3.63
Model resolution range (Å)	4.4 – ∞	4.1 – ∞	4.3 – ∞	-	-	3.2 – ∞	-	4.0 – ∞	3.4 – ∞	3.6 – ∞
CC model vs. data	0.79	0.79	0.76	-	-	0.81	-	0.77	0.81	0.79
Atoms (excluding H)	67,680	74,232	140,004	-	-	72,732	-	71,054	65,550	73,062
Protein residues	8,376	9,126	17,562	-	-	8,400	-	8,511	8,142	9,354
Ligands	ZN: 12	ZN: 6	ZN: 6	-	-	ZN: 12	-	-	ZN: 6	-
Mean protein B-factor ($Å^2$)	62.46	68.71	110.16	-	-	46.62	-	108.08	44.55	79.28
Mean DNA B-factor ($Å^2$)	-	-	-	-	-	159.62	-	264.01	-	-
Mean ligand B-factor ($Å^2$)	55.62	63.78	83.91	-	-	46.23	-	-	46.21	-
Bond length RMSD (Å)	0.004	0.004	0.004	-	-	0.004	-	0.004	0.005	0.004
Bond angle RMSD (°)	0.951	0.965	0.909	-	-	1.033	-	0.682	1.091	1.076
**Model validation**										
MolProbity score	0.77	0.96	0.98	-	-	0.81	-	0.79	0.76	0.80
Clash score	0.89	1.74	1.81	-	-	1.07	-	0.97	0.86	1.03
Ramachandran favored (%)	98.55	97.87	97.85	-	-	98.55	-	98.57	99.03	99.09
Ramachandran allowed (%)	1.45	2.13	2.15	-	-	1.45	-	1.43	0.97	0.91
Ramachandran disallowed (%)	0.00	0.00	0.00	-	-	0.00	-	0.00	0.00	0.00
Rotamer outliers (%)	0.00	0.00	0.00	-	-	0.00	-	0.00	0.00	0.00
Cβ outliers (%)	0.00	0.00	0.00	-	-	0.00	-	0.00	0.00	0.00
DNAtco confal	-	-	-	-	-	59	-	63	-	-
DNAtco step RMSD > 0.5 Å (%)	-	-	-	-	-	2.94	-	1.61	-	-
**PDB entry ID**	8QJE	8QKH	8R5G	-		8Q7D	-	8QEM	8Q01	8Q1I

Table S2. Annotation of phage 812 gene products characterized in this work.

**Phage species**	**NCBI taxon ID**	**ICTV 2022 taxonomy** (Class; Family; Subfamily; Genus)		
Staphylococcus phage 812 K1/420	307898	*Caudoviricetes; Herelleviridae; Twortvirinae; Kayvirus*		
**Protein name**	**Gene product**	**Residue length**	**MW (kDa)**	**Uniprot ID**
terminator decoration protein	gp56	87	10.1	A0A0U1WZ69
portal protein	gp91	563	64.1	A0A0U1WIV9
adaptor protein	gp96	302	34.2	A1YTN6
stopper protein	gp97	292	33.7	A1YTN7
tail terminator protein	gp99	278	31.8	A1YTN9
tail sheath protein	gp101	587	64.5	A0A0U1WZ79
tail tube protein	gp102	142	13.1	A1YTP2
tail tape measure protein	gp109	1352	143.9	A0A0U1X2G0
stopper decoration protein	gp164	152	17.9	A0A0U1WIM1

Table S3. Annotation of subdomains of phage 812 neck and tail proteins.

**Protein name**	**Subdomain**	**Residue range**
terminator decoration protein	terminal strands	3 - 17, 70 - 84
	central domain	18 - 69
portal protein	wing	40 - 263, 404 - 431
	stem	264 - 285, 343 - 363
	clip	286 - 342
	tunnel loop	375 - 392
	crown base	432 - 437
	crown funnel	439 - 503
	crown globules	508 - 563
adaptor protein	α-helical core	49 - 91, 219 - 241, 273 - 296
	β-hairpin	249 - 264
	channel loop	265 - 272
	β-sandwich	96 - 205
stopper protein	β-barrel	56 - 61, 93 - 101, 119 - 138, 210 - 218, 241 - 245
	tetra-cysteine loop	63 - 90
	gating loop	104 - 118
	β-sandwich	143 - 208
tail terminator protein	β-sandwich	98 - 170
	extended C-terminus	262 - 278
tail sheath protein	domain I	483 - 587
	domain II	2 - 94, 316 - 482
	domain III	95 - 150, 251 - 315
	domain IV	151 - 250
stopper decoration protein	N-terminal domain	1 - 95
	C-terminal domain	96 - 152

Table S4. Atomic models built into cryo-EM reconstructions. Reported values are numbers of built residues per chain (in parentheses, number of built chains per particle). Refined models are in black, models flexibly fitted to density in blue, and models rigidly fitted in gray. For decoration and tail proteins with multiple rings of symmetrically-unrelated conformations, the values for individual rings are listed on successive rows, in an order following the capsid-to-tail direction.

	phage 812 virion						phage 812 genome release intermediate					
**Cryo-EM reconstruction**	**neck C12**	**neck C6**	**neck/tail C6**	**neck/tail C3**	**neck/tail C1**	**composite C6**	**neck C12**	**anchor DNA C1**	**channel DNA C1**	**neck C6**	**neck/tail C6**	**composite C6**
**Atomic model**												
terminator decoration	-	86 (6),<br>82 (6)*	86 (6),<br>82 (6)*	-	-	86 (6),<br>82 (6)*	-	-	-	-	-	-
portal protein	439 (12)	-	-	-	-	439 (12)	441 (12)	441 (12)	450 (12)[$]	-	-	441 (12)
adaptor protein	259 (12)	259 (12)	259 (12)	-	-	259 (12)	259 (12)	259 (12)	259 (12)	259 (12)	-	259 (12)
stopper protein	-	268 (6)	268 (6)	-	-	268 (6)	-	-	-	227 (6)	-	227 (6)
tail terminator protein	-	275 (6)	275 (6)	275 (6)	275 (6)	275 (6)	-	-	-	277 (6)	277 (6)	277 (6)
tail sheath protein	-	-	562 (6)***,<br>562 (6)***<br>-	361 (6),<br>361 (4 × 6)<br>-	361 (6),<br>361 (4 × 6)<br>-	562 (6)***,<br>562 (6)***<br>-	-	-	-	56 (6)	352 (6),<br>367 (6),<br>563 (6)[%]	352 (6),<br>367 (6),<br>563 (6)[%]
tail tube protein	-	-	141 (6),<br>141 (6)	141 (6),<br>141 (5 × 6)	141 (6),<br>141 (5 × 6)	141 (6),<br>141 (6)	-	-	-	139 (6),<br>140 (6)	-	139 (6),<br>140 (6)
tail tape measure protein	-	-	-	14 (3)	14 (3)	-	-	-	-	-	-	-
stopper decoration protein	-	148 (6)**,<br>144 (6)	148 (6)**,<br>144 (6)	-	-	148 (6)**,<br>144 (6)	-	-	-	-	-	-
anchor DNA	-	-	-	-	-	-	120 (2)	139 (2)	-	-	-	-
channel DNA	-	-	-	-	-	-	-	-	63 (2)	-	-	-
tail tube B-DNA	-	-	-	-	32 (2)	-	-	-	-	-	-	-
**PDB ID**	8QJE	8QKH	8R5G	N.D.[#]	N.D.	8R69	8Q7D	N.D.	8QEM	8Q01	8Q1I	8QEK

Note 1: predicted atomic models were fitted to densities and refined without rebuilding for the following protein domains: terminator decoration protein res. 28-58,63-68*, stopper decoration protein res. 94-152**, native tail sheath res. 95-273,294-315***, contracted tail sheath res. 96-271,295-314[%].

Note 2: [#]Not deposited, only for the purpose of figures.

Note 3: [$]The twelve portal chains differ in the lengths of built tunnel loops and therefore in the total number of built residues; we report here the average number of residues.

Table S5. Interactions between portal complex and DNA rings in virion and genome release intermediate. Distance *d* reports the minimal distance between a phosphate oxygen of a B-form circular dsDNA model and a charged nitrogen of an Arg or Lys side chain of the portal dodecamer.

**Particle type**	**dsDNA ring radius (Å)**	**modelled DNA length (bp)**	$d \leq 4$ **Å**	**4 Å <** $d \leq 6$ **Å**
virion	52	94	Arg137, Lys465	Lys138
	71	131	Lys138, Lys194	Arg145, Lys425
	85	155	Lys189, Lys192, Lys212, Lys221	
genome release intermediate	65	120	Arg137, Arg145, Lys425	Lys194

Table S6. X-ray data refinement and validation statistics for stopper protein gp97 (PDB ID 8QGR).

**Data collection**			**Refinement**	
Space group	P $6_1 2_1 2_1$ (177)		Resolution (Å)	37.13 - 2.20
Cell dimensions:			No. of reflections	194,930
a, b, c (Å)	93.37, 93.37, 122.53		No. of unique reflections	16,664 (1,627)
α, β, γ (°)	90, 90, 120		R_{work} / R_{free}	0.190 / 0.219
Resolution (Å)	37.13 - 2.20 (2.28 - 2.20)*		Built residues	41 - 223, 237 - 260, 263 - 276 (total 221, 75.7%)
R_{merge}	0.130 (0.689)		No. of atoms (excluding H):	
I/σI	23.26 (4.21)		protein	1815
Completeness (%)	99.9 (99.8)		ion	1
Redundancy (%)	11.70		water	146
NCS	none		B-factors:	
			protein	34.00
			ion	23.83
			water	35.80
			RMSD:	
			bond lengths (Å)	0.007
			bond angles (°)	1.333
			MolProbity score	1.19
			Clash score	3.31
			Poor rotamers	1 (0.49 %)
			Ramachandran plot:	
			Disallowed (%)	0.00
			Allowed (%)	2.33
			Favored (%)	97.67

*Data collected on one crystal. Values in parentheses are for the highest-resolution shell.

Table S7. Comparison of stopper protein conformations in closed (X-ray) and open (virion) state. The conformations aligned as hexamers have a high overall structural divergence, with a modest similarity in the β-barrel subdomain. Alignment on β-barrel monomers shows that the β-barrels and the tetra-cysteine loops have largely the same local structures, meaning they rearrange from the closed to the open state as rigid bodies. Other subdomains (in particular the gating loop and the C-terminus) experience a local conformational change in addition to the global rearrangement, which manifests as high overall structural divergence between the aligned monomers. The pairwise matched residues are res. 40-223, 237-269 for the monomers, res. 56-61, 93-100, 119-138, 210-218, 241-245 for β-barrels, res. 62-92 for tetra-cysteine loops, and res. 56-100, 119-138, 210-218, 241-245 for β-barrels including tetra-cysteine loops.

X-ray stopper aligned to virion stopper on	Pairwise Cα-RMSD ($Å^2$) from X-ray protein to			
	virion monomer (217 res.)	virion β-barrel + tetra-Cys loop (79 res.)	virion β-barrel (48 res.)	virion tetra-Cys loop (31 res.)
**hexamer**	15.56	4.16	2.70	5.73
**β-barrel monomer**	14.32	1.36	1.26	1.50

Table S8. Hydrogen bonds between portal protein and anchor DNA bases. Estimated hydrogen bond lengths between the Lys138 NZ donor and acceptors at the minor groove edges of the two closest base pairs, for the 16 nucleotide combinations of these Watson-Crick base pairs. Green fields mark H-bond lengths < 4.0 Å.

5'- F5pF6 -3'			H-bond length (Å; mean ± sd, n=3 simulation runs)	
**dinucleotide**	**pYrimidine / puRine**	**Weak / Strong base pair**	**to F6 N3|O2**	**to R6 N3|O2**
**AT**	RY	WW	3.60 ± 0.07	3.08 ± 0.06
**GT**	RY	SW	3.71 ± 0.05	3.53 ± 0.08
**AC**	RY	WS	3.78 ± 0.04	2.97 ± 0.01
**GC**	RY	SS	4.17 ± 0.04	3.88 ± 0.20
**AA**	RR	WW	4.06 ± 0.14	3.05 ± 0.06
**GA**	RR	SW	4.14 ± 0.07	3.32 ± 0.24
**AG**	RR	WS	4.44 ± 0.09	3.32 ± 0.19
**GG**	RR	SS	4.55 ± 0.08	3.59 ± 0.02
**TT**	YY	WW	3.99 ± 0.09	3.81 ± 0.06
**CT**	YY	SW	3.73 ± 0.04	3.93 ± 0.03
**TC**	YY	WS	4.36 ± 0.01	3.78 ± 0.13
**CC**	YY	SS	4.16 ± 0.16	3.84 ± 0.04
**TA**	YR	WW	4.42 ± 0.02	3.57 ± 0.20
**CA**	YR	SW	4.24 ± 0.15	4.00 ± 0.18
**TG**	YR	WS	4.70 ± 0.08	3.52 ± 0.04
**CG**	YR	SS	4.57 ± 0.09	4.17 ± 0.04

Table S9. Effect of base type on hydrogen bond length between portal protein and anchor DNA. Hydrogen bond lengths (Å) from Lys138 to base F6 or R6 (Table S8) are cross-tabulated according to the base ring (R or Y) and the base pair (S or W) at the nucleotide position. The H-bond length decreases on the red to green color scale. The estimated average effect of a pyrimidine instead of a purine on the H-bond length to F6 is -0.45 Å (two-sided *t-test* with n1=n2=8, 14 d.o.f., 95% CI: -0.63, -0.27, p-val=1.2×10^{-4}) and the effect of a weak instead of a strong base pair is -0.36 Å (two-sided *t-test* with n1=n2=8, 14 d.o.f., 95% CI: -0.54, -0.17, p-val=9.8×10^{-4}). For the H-bond to R6, the effect is -0.49 Å (two-sided *t-test* with n1=n2=8, 14 d.o.f., 95% CI: -0.67, -0.30, p-val=7.3×10^{-5}) for a pyrimidine and -0.40 Å (two-sided *t-test* with n1=n2=8, 14 d.o.f., 95% CI: -0.58, -0.21, p-val=4.6×10^{-4}) for a weak base pair.

**Base F6:**	puRine	pYrimidine	effect Y-R		**Base R6:**	puRine	pYrimidine	effect Y-R
Strong	4.57 ± 0.11	4.12 ± 0.24	-0.45		Strong	3.99 ± 0.14	3.58 ± 0.23	-0.41
Weak	4.22 ± 0.16	3.76 ± 0.17	-0.46		Weak	3.67 ± 0.15	3.11 ± 0.15	-0.57
effect W-S	-0.35	-0.36			effect W-S	-0.32	-0.48	

Table S10. *Herelleviridae* species compared in Figs. S8 and S13.

**Species**	**Portal protein NCBI ID**	**Stopper protein NCBI ID**	**Taxon NCBI ID**	**Taxonomic classification** (Family; Subfamily; Genus)
Staphylococcus virus K	YP_009041312.1	YP_009041318.1	221915	*Herelleviridae; Twortvirinae; Kayvirus*
Staphylococcus phage phiIBB-SEP1	YP_009600933.1	YP_009600939.1	1340769	*Herelleviridae; Twortvirinae; Sepunavirus*
Staphylococcus phage phiSA_BS1	YP_009799684.1	YP_009799513.1	2126734	*Herelleviridae; Twortvirinae; Baoshanvirus*
Staphylococcus virus SA11	YP_007005624.1, YP_007005625.1	YP_007005617.1	1204543	*Herelleviridae; Twortvirinae; Silviavirus*
Staphylococcus phage vB_SscM-1	YP_009786245.1	YP_009786251.1	1868844	*Herelleviridae; Twortvirinae; Sciuriunavirus*
Staphylococcus virus Twort	YP_238542.1	YP_238549.1	55510	*Herelleviridae; Twortvirinae; Twortvirus*
Enterococcus virus ECP3	YP_009147103.1	YP_009147096.1	1498168	*Herelleviridae; Brockvirinae; Kochikohdavirus*
Enterococcus virus EFDG1	YP_009218320.2	YP_009218328.1	1597976	*Herelleviridae; Brockvirinae; Schiekvirus*
Listeria phage vB_LmoM_AG20	YP_007676742.1	YP_007676748.1	1168744	*Herelleviridae; Jasinkavirinae; Pecentumvirus*
Bacillus phage phiAGATE	YP_007349202.1	YP_007349196.1	1204533	*Herelleviridae; Bastillevirinae; Agatevirus*
Bacillus virus Bastille	YP_006907304.1	YP_006907311.1	57477	*Herelleviridae; Bastillevirinae; Bastillevirus*
Bacillus phage B4	YP_006908499.1	YP_006908492.1	1141133	*Herelleviridae; Bastillevirinae; Bequatrovirus*
Bacillus phage vB_BceM_Bc431v3	YP_007677133.1	YP_007677127.1	1195072	*Herelleviridae; Bastillevirinae; Caeruleovirus*
Bacillus phage Eldridge	YP_009274760.1	YP_009274766.1	1776293	*Herelleviridae; Bastillevirinae; Eldridgevirus*
Bacillus phage vB_BmeM-Goe8	YP_009850005.1	YP_009850011.1	2593638	*Herelleviridae; Bastillevirinae; Goettingenvirus*
Bacillus phage vB_BsuM-Goe3	YP_009832049.1	YP_009832055.1	1933063	*Herelleviridae; Bastillevirinae; Grisebachstrassevirus*
Bacillus phage BSP38	YP_009840537.1	YP_009840544.1	2283013	*Herelleviridae; Bastillevirinae; Jeonjuvirus*
Bacillus phage Mater	YP_009151142.1	YP_009151136.1	1540090	*Herelleviridae; Bastillevirinae; Matervirus*
Bacillus phage Moonbeam	YP_009151616.1	YP_009151622.1	1540091	*Herelleviridae; Bastillevirinae; Moonbeamvirus*
Bacillus phage Grass	YP_008771433.1	YP_008771438.1	1406785	*Herelleviridae; Bastillevirinae; Nitunavirus*
Bacillus phage Shbh1	YP_009275280.1	YP_009275286.1	1796992	*Herelleviridae; Bastillevirinae; Shalavirus*
Bacillus phage SIOphi	YP_009625668.1	YP_009625662.1	1285382	*Herelleviridae; Bastillevirinae; Siophivirus*
Bacillus virus TsarBomba	YP_009206889.1	YP_009206896.1	1690456	*Herelleviridae; Bastillevirinae; Tsarbombavirus*
Bacillus phage W.Ph.	YP_004957027.1	YP_004957033.1	764595	*Herelleviridae; Bastillevirinae; Wphvirus*
Bacillus phage CampHawk	YP_008770006.1	YP_008770012.1	1406783	*Herelleviridae; Spounavirinae; Okubovirus*
Bacillus phage CP-51	YP_009099060.1	YP_009099066.1	1391188	*Herelleviridae; Spounavirinae; Siminovitchvirus*
Lactobacillus phage LpeD	YP_009835298.1	YP_009835302.1	2041210	*Herelleviridae; –; Elpedvirus*
Lactobacillus phage Bacchae	YP_009798045.1	YP_009798050.1	2079429	*Herelleviridae; –; Harbinvirus*
Lactobacillus phage 521B	YP_009843933.1	YP_009843940.1	2510942	*Herelleviridae; –; Tybeckvirus*
Lactobacillus phage LfeInf	YP_009222296.1	YP_009222303.1	1567484	*Herelleviridae; –; Hopescreekvirus*
Lactobacillus virus Lb338-1	YP_002790785.1	YP_002790791.1	632112	*Herelleviridae; –; Mooreparkvirus*
Lactobacillus virus LP65	YP_164747.1	YP_164742.1	298338	*Herelleviridae; –; Salchichonvirus*
Lactobacillus phage 3-521	YP_009844052.1	YP_009844061.1	2510943	*Herelleviridae; –; Watanabevirus*

Table S11. Portal complexes reported in Fig. S9.

**Species**<br>**ICTV 2022 taxonomic classification** *(Class; Order; Family; Subfamily; Genus)*	**Taxon NCBI ID**	**PDB ID**	**Reference**
Human alphaherpesvirus 1<br>*Herviviricetes; Herpesvirales; Herpesviridae; Alphaherpesvirinae; Simplexvirus*	10298	6od7	Liu *et al.* (2019), Nature[2]
Escherichia phage T4<br>*Caudoviricetes; —; Straboviridae; Tevenvirinae; Tequatrovirus*	2681598	3ja7	Sun *et al.* (2015), Nat. Commun.[3]
Thermus phage P23-45 (G20)<br>*Caudoviricetes; —; —; —; Oshimavirus*	2914006	6ibg	Bayfield *et al.* (2019), Proc. Natl. Acad. Sci.[4]
Bacillus phage phi29<br>*Caudoviricetes; —; Salasmaviridae; Picovirinae; Salasvirus*	2884424	6qym	Xu *et al.* (2019), Nat. Commun.[5]
Escherichia phage T7<br>*Caudoviricetes; —; Autographiviridae; Studiervirinae; Teseptimavirus*	10760	7bou	Chen *et al.* (2020), Protein Cell[6]
Salmonella phage P22<br>*Caudoviricetes; —; —; —; Lederbergvirus*	2908168	4v4k	Olia *et al.* (2011), Nat. Struct. Mol. Biol.[7]
Staphylococcus phage P68<br>*Caudoviricetes; —; Rountreeviridae; Rakietenvirinae; Rosenblumvirus*	204090	6q3g	Hrebík *et al.* (2019), Sci. Adv.[8]
Rhodobacter capsulatus DE442 (GTA)<br>*Alphaproteobacteria; Rhodobacterales; Paracoccaceae; —; Rhodobacter*	1415160	6te8	Bárdy *et al.* (2020), Nat. Commun.[9]

Supplementary References

1. Orlov, I. *et al.* CryoEM structure and assembly mechanism of a bacterial virus genome gatekeeper. *Nat. Commun.* **13**, 7283 (2022).
2. Liu, Y.-T., Jih, J., Dai, X., Bi, G.-Q. & Zhou, Z. H. Cryo-EM structures of herpes simplex virus type 1 portal vertex and packaged genome. *Nature* **570**, 257–261 (2019).
3. Sun, L. *et al.* Cryo-EM structure of the bacteriophage T4 portal protein assembly at near-atomic resolution. *Nat. Commun.* **6**, 7548 (2015).
4. Bayfield, O. W. *et al.* Cryo-EM structure and in vitro DNA packaging of a thermophilic virus with supersized T=7 capsids. *Proc. Natl. Acad. Sci.* **116**, 3556–3561 (2019).
5. Xu, J., Wang, D., Gui, M. & Xiang, Y. Structural assembly of the tailed bacteriophage φ29. *Nat. Commun.* **10**, 2366 (2019).
6. Chen, W. *et al.* Structural changes of a bacteriophage upon DNA packaging and maturation. *Protein Cell* **11**, 374–379 (2020).
7. Olia, A. S., Prevelige, P. E., Johnson, J. E. & Cingolani, G. Three-dimensional structure of a viral genome-delivery portal vertex. *Nat. Struct. Mol. Biol.* **18**, 597–603 (2011).
8. Hrebík, D. *et al.* Structure and genome ejection mechanism of Staphylococcus aureus phage P68. *Sci. Adv.* **5**, eaaw7414 (2019).
9. Bárdy, P. *et al.* Structure and mechanism of DNA delivery of a gene transfer agent. *Nat. Commun.* **11**, 3034 (2020).
