# Supplementary material for: Genome anchoring, retention, and release by neck proteins of Staphylococcus phage 812
Source: Commun Biol. 2026 Jan 8;9:199. doi: 10.1038/s42003-025-09477-8 (PMC12886913; doi:10.1038/s42003-025-09477-8)
Supplement: Supplementary file 3 — Description of Additional Supplementary Files [file 42003_2025_9477_MOESM3_ESM.pdf]

Description of Additional Supplementary File

File name: Supplementary Data 1

Description: The source data behind the graphs in the main manuscript and in Supplementary Information.pdf (except for those included in repositories).

File name: Supplementary Data 2

Description: Processed phage particle mass-spectrometry data.
