# Supplementary material for: Genome anchoring, retention, and release by neck proteins of Staphylococcus phage 812
Source: Commun Biol. 2026 Jan 8;9:199. doi: 10.1038/s42003-025-09477-8 (PMC12886913; doi:10.1038/s42003-025-09477-8)
Supplement: Supplementary file 6 — Reporting summary [file 42003_2025_9477_MOESM6_ESM.pdf]

nature portfolio

Corresponding author(s): Pavel Plevka

Last updated by author(s): Dec 4, 2025

Reporting Summary

Nature Portfolio wishes to improve the reproducibility of the work that we publish. This form provides structure for consistency and transparency in reporting. For further information on Nature Portfolio policies, see our Editorial Policies and the Editorial Policy Checklist.

Statistics

For all statistical analyses, confirm that the following items are present in the figure legend, table legend, main text, or Methods section.

n/a	Confirmed	
☐	☒	The exact sample size (*n*) for each experimental group/condition, given as a discrete number and unit of measurement
☐	☒	A statement on whether measurements were taken from distinct samples or whether the same sample was measured repeatedly
☐	☒	The statistical test(s) used AND whether they are one- or two-sided<br>*Only common tests should be described solely by name; describe more complex techniques in the Methods section.*
☒	☐	A description of all covariates tested
☒	☐	A description of any assumptions or corrections, such as tests of normality and adjustment for multiple comparisons
☒	☐	A full description of the statistical parameters including central tendency (e.g. means) or other basic estimates (e.g. regression coefficient) AND variation (e.g. standard deviation) or associated estimates of uncertainty (e.g. confidence intervals)
☐	☒	For null hypothesis testing, the test statistic (e.g. *F*, *t*, *r*) with confidence intervals, effect sizes, degrees of freedom and *P* value noted<br>*Give P values as exact values whenever suitable.*
☒	☐	For Bayesian analysis, information on the choice of priors and Markov chain Monte Carlo settings
☒	☐	For hierarchical and complex designs, identification of the appropriate level for tests and full reporting of outcomes
☒	☐	Estimates of effect sizes (e.g. Cohen's *d*, Pearson's *r*), indicating how they were calculated

Our web collection on statistics for biologists contains articles on many of the points above.

Software and code

Policy information about availability of computer code

Data collection	SerialEM 3.7.14, Gctf 1.0.6, MotionCor2 1.4.0, MinKNOW 23.11.4
Data analysis	EMAN2, Gautomatch 0.56, Relion 3.1, xmipp 3.0, R 4.1.2, Coot 0.9, Phenix 1.19, Isolde 1.4, XDS version 2016-11-01, CCP4 Suite 8.0, Phaser 2.8.3, RefMac5 5.8, UCSF Chimera 1.15, UCSF ChimeraX 1.9, FastQC 0.11.8, Trimmomatic Galaxy 0.39, RASTtk, Unicycler Galaxy 0.5.0, Geneious Prime 2025.0.3, Proteome Discoverer 1.4, Mascot 2.6.2

For manuscripts utilizing custom algorithms or software that are central to the research but not yet described in published literature, software must be made available to editors and reviewers. We strongly encourage code deposition in a community repository (e.g. GitHub). See the Nature Portfolio guidelines for submitting code & software for further information.

Data

Policy information about availability of data

All manuscripts must include a data availability statement. This statement should provide the following information, where applicable:

- Accession codes, unique identifiers, or web links for publicly available datasets
- A description of any restrictions on data availability
- For clinical datasets or third party data, please ensure that the statement adheres to our policy

The GenBank/ENA/DDBJ accession number of the phage 812 strain K1/420 genome is KJ206563.2. Raw data from phage DNA sequencing was deposited in NCBI under the BioProject ID PRJNA1269267. Cryo-EM density maps are available in the Electron Microscopy Data Bank under accession IDs EMD-18385, EMD-18395,

EMD-18412, EMD-18489, EMD-18516, and EMD-18919. Atomic coordinates and X-ray structure factors are available in the Protein Data Bank under accession IDs 8Q01, 8Q1I, 8Q7D, 8QEM, 8QEK, 8QGR, 8QJE, 8QKH, 8R5G, and 8R69. Mass spectrometry data were deposited to PRIDE repository under identifier PXD071586.

Research involving human participants, their data, or biological material

Policy information about studies with human participants or human data. See also policy information about sex, gender (identity/presentation), and sexual orientation and race, ethnicity and racism.

Reporting on sex and gender	This research did not involve human participants nor their data or biological material.
Reporting on race, ethnicity, or other socially relevant groupings	This research did not involve human participants nor their data or biological material.
Population characteristics	This research did not involve human participants nor their data or biological material.
Recruitment	This research did not involve human participants nor their data or biological material.
Ethics oversight	This research did not involve human participants nor their data or biological material.

Note that full information on the approval of the study protocol must also be provided in the manuscript.

Field-specific reporting

Please select the one below that is the best fit for your research. If you are not sure, read the appropriate sections before making your selection.

☒ Life sciences ☐ Behavioural & social sciences ☐ Ecological, evolutionary & environmental sciences

For a reference copy of the document with all sections, see nature.com/documents/nr-reporting-summary-flat.pdf

Life sciences study design

All studies must disclose on these points even when the disclosure is negative.

Sample size	For cryo-EM data collection, 30,553 resp. 15,371 images were acquired on two independent sample preparations, corresponding to 23,947 images of individual virion particles and 25,413 of images of individual phages after tail contraction. For X-ray crystallography, data were collected on one crystal. For full phage genome sequencing, two independent samples were used and full genome coverage was above 600x and 3000x. For residual genome sequencing, 332,896 sequencing reads were collected.
Data exclusions	For cryo-EM, images were quality-filtered based on artifacts and resolution. Individual phage particle images were quality-filtered and classified into categories based on genome content and symmetry. For genome sequencing, bases with quality below 20 were trimmed. For residual DNA sequencing, reads with length above 8,484 bp were excluded (7% of reads).
Replication	For full genome sequencing, reads were sequenced on two platforms, with consistent result.
Randomization	For reconstruction and resolution estimation of cryo-EM maps, particle datasets were randomly split into two subsets equal in size and reconstructed independently. For genome sequencing, reads with multiple best matches were mapped to the genome randomly.
Blinding	Initial virion particle selection in cryo-EM was performed manually based on the integrity of the phage particle and presence of genome; for particles after genome release treatment the selection was done using an automated algorithm. In subsequent steps all particles were filtered and categorized using automated procedures.

Reporting for specific materials, systems and methods

We require information from authors about some types of materials, experimental systems and methods used in many studies. Here, indicate whether each material, system or method listed is relevant to your study. If you are not sure if a list item applies to your research, read the appropriate section before selecting a response.

Materials & experimental systems

n/a	Involved in the study
☒	☐ Antibodies
☒	☐ Eukaryotic cell lines
☒	☐ Palaeontology and archaeology
☒	☐ Animals and other organisms
☒	☐ Clinical data
☒	☐ Dual use research of concern
☒	☐ Plants

Methods

n/a	Involved in the study
☒	☐ ChIP-seq
☒	☐ Flow cytometry
☒	☐ MRI-based neuroimaging

Plants

Seed stocks	*Report on the source of all seed stocks or other plant material used. If applicable, state the seed stock centre and catalogue number. If plant specimens were collected from the field, describe the collection location, date and sampling procedures.*
Novel plant genotypes	*Describe the methods by which all novel plant genotypes were produced. This includes those generated by transgenic approaches, gene editing, chemical/radiation-based mutagenesis and hybridization. For transgenic lines, describe the transformation method, the number of independent lines analyzed and the generation upon which experiments were performed. For gene-edited lines, describe the editor used, the endogenous sequence targeted for editing, the targeting guide RNA sequence (if applicable) and how the editor was applied.*
Authentication	*Describe any authentication procedures for each seed stock used or novel genotype generated. Describe any experiments used to assess the effect of a mutation and, where applicable, how potential secondary effects (e.g. second site T-DNA insertions, mosiacism, off-target gene editing) were examined.*
